# High Consistency of Structure-Based Design and X-Ray Crystallography: Design, Synthesis, Kinetic Evaluation and Crystallographic Binding Mode Determination of Biphenyl-*N*-acyl-β-d-Glucopyranosylamines as Glycogen Phosphorylase Inhibitors

**DOI:** 10.3390/molecules24071322

**Published:** 2019-04-03

**Authors:** Thomas Fischer, Symeon M. Koulas, Anastasia S. Tsagkarakou, Efthimios Kyriakis, George A. Stravodimos, Vassiliki T. Skamnaki, Panagiota G.V. Liggri, Spyros E. Zographos, Rainer Riedl, Demetres D. Leonidas

**Affiliations:** 1 Institute of Chemistry and Biotechnology, Center of Organic and Medicinal Chemistry, Zurich University of Applied Sciences, Einsiedlerstrasse 31, 8820 Wädenswil, Switzerland; thomas.fischer@zhaw.ch; 2 Department of Biochemistry and Biotechnology, University of Thessaly, Biopolis, 41500 Larissa, Greece; sym.koulas@gmail.com (S.M.K.); anastasiatsagk@hotmail.com (A.S.T.); kyriakis.ef@gmail.com (E.K.); stravodimos@windowslive.com (G.A.S.); vskamnaki@bio.uth.gr (V.T.S.); b_liggri@windowslive.com (P.G.V.L.); 3 Institute of Biology, Pharmaceutical Chemistry and Biotechnology, National Hellenic Research Foundation, 48 Vassileos Constantinou Avenue, 11635 Athens, Greece; sez@eie.gr

**Keywords:** structure-based design, glycogen phosphorylase inhibitor, glycogen metabolism, type 2 diabetes, X-ray crystallography, *N*-acyl-β-d-glucopyranosylamine

## Abstract

Structure-based design and synthesis of two biphenyl-*N*-acyl-β-d-glucopyranosylamine derivatives as well as their assessment as inhibitors of human liver glycogen phosphorylase (hlGPa, a pharmaceutical target for type 2 diabetes) is presented. X-ray crystallography revealed the importance of structural water molecules and that the inhibitory efficacy correlates with the degree of disturbance caused by the inhibitor binding to a loop crucial for the catalytic mechanism. The in silico-derived models of the binding mode generated during the design process corresponded very well with the crystallographic data.

## 1. Introduction

**Figure 1:**
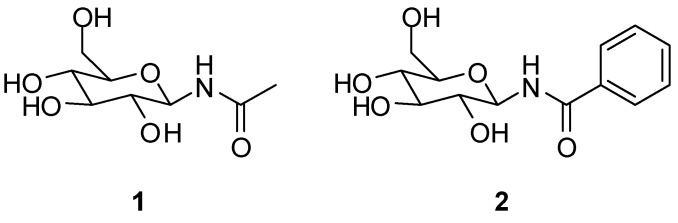
Previously described glucopyranosyl containing GP inhibitors **1** and **2**.

Glycogen phosphorylase (GP) is a validated pharmaceutical target for the discovery of new antidiabetic drugs [1,2]. GP is an allosteric enzyme that catalyses the first step of glycogen degradation in the liver and muscle to produce glucose-1-phosphate. The human hepatic enzyme (hlGPa) is responsible to maintain blood glucose homeostasis [3]. GP senses plasma levels of glucose to ensure that glycogenolysis is halted when glucose is abundant in plasma. This is achieved through allosteric binding of glucose to GP and stabilization of a conformation that enables the inactivation of the enzyme [4]. However, glucose is not a potent inhibitor of GP (*K*_i_ = 3.2 mM) [5] since blood glucose levels have to be maintained. Thus, the development of potent GP inhibitors was based on glucose derivatives by modifying glucose to generate inhibitors with better binding affinity [6]. Early structural studies proposed the modification of the β-1 position and the addition of suitable hydrophobic groups to exploit interactions with a hydrophobic pocket [7,8] in the direction of the β-anomeric substituent, adjacent to the active site formed mostly by hydrophobic residues (termed as the β-channel) [9]. A –CONH– linker between glucopyranose and the hydrophobic groups has proven to be particularly efficient since NH forms a hydrogen bond to the side chain of His377 [6]. One of the first attempts to designing potent GP inhibitors was to add a methyl or a phenyl group (**1** and **2**, Figure 1) that led to a 100- and 39.5-fold decrease of the *K*_i_ value, respectively compared to that of glucose [7].

Here, we present the structure-based design and organic synthesis of two new GP glucopyranosyl inhibitors as well as the kinetic and X-ray crystallography assessment of the new compounds.

## 2. Results

### 2.1. Molecular Modelling

**Figure 2:**
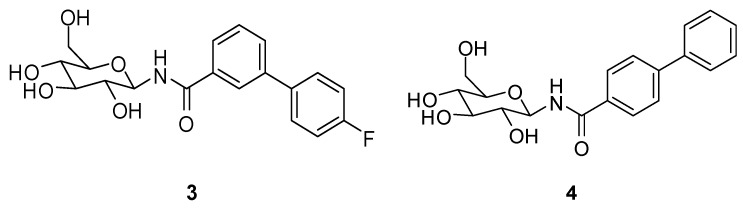
Structure-based designed GP inhibitors containing a *para* fluorinated phenyl group in the *meta* position (**3**), and with a phenyl residue in *para* position (**4**).

We designed the two molecules **3** and **4,** as depicted in Figure 2, as potential inhibitors of GP.

**Figure 3:**
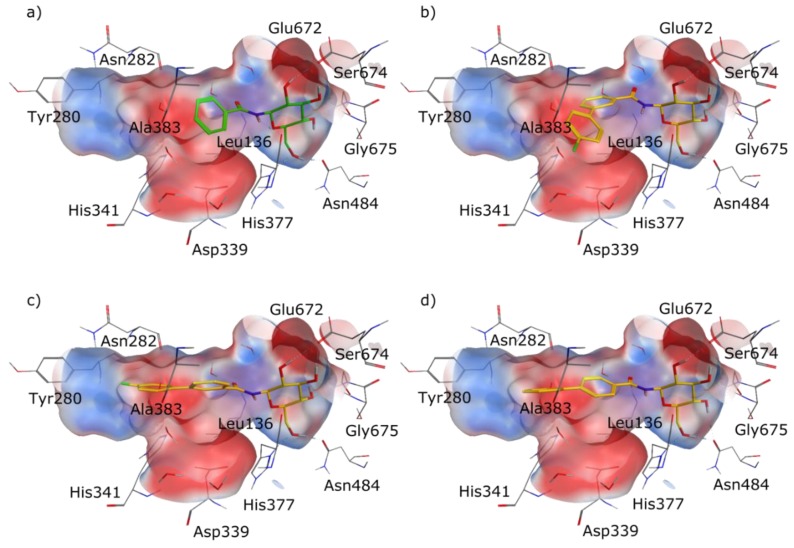
(**a**) Co-crystal structure PDB 3G2N; (**b**) compound **3** docked to PDB 3G2N (highest scored pose; −9.1160); (**c**) compound **3** docked to PDB 3G2N (second highest scored pose; −8.9981); and (**d**) compound **4** docked to PDB 3G2N (highest scored pose; −9.4502), co-crystallized ligand with carbons in green, docked ligands with carbons in yellow.

For the structure-based design of novel GP inhibitors, we examined the co-crystal structure of **2** bound to GP (PDB entry 3G2N) [9]. As is visible in Figure 3a, the glucopyranose moiety forms a network of hydrogen bonds with the amino acid residues His377, Asn484, Glu672 and Gly675 whereas the phenyl group points into a spacious cavity (β-channel) that provides sufficient room for further ligand growing. We hypothesized that an aromatic ring attached in *para* position of the phenyl group can interact with His341 by aromatic interactions for inhibitor enhancement. The 4-fluorophenyl unit in the *meta* position was intended to populate the β-channel and engage in constructive interactions with the amino acids Asn282 and His341 in close proximity. The strategic placement of a single fluorine atom is reported to have also positive effects on the pharmacokinetic profile of drug molecules, and by the formation of hydrogen bonds it can contribute to elevated inhibitor potency [10,11,12,13,14]. We performed docking experiments applying pharmacophore queries for the conservation of the hydrogen bonds formed by the glucopyranose residue and to maintain optimal ligand orientation.

Both postulated inhibitors could be docked into the cavity with the glucopyranose head group being anchored to fulfil the pharmacophore query. Compound **3** displayed two almost equally ranked docking poses that differ significantly in orientation by populating two opposite sites within the cavity. In the highest ranked pose (Figure 3b) the fluorine atom points towards the amino acids Asp339 and Ala383 and might form a water mediated interaction with one of the solvent molecules present in close proximity. In the second highest ranked docking pose, represented in Figure 3c, the fluorine penetrates the cavity towards Tyr280. In this pose, the fluorine containing phenyl ring overlaps with a water molecule that is present in the crystal structure PDB 3G2N which has to be replaced by the incoming ligand (solvent was set to inactive in the force field for the docking experiment). For compound **4** only one highly ranked orientation was obtained in the molecular modeling (Figure 3d) with the ligand being oriented in a straight line. We decided to synthesize the designed compounds to examine their inhibitory potential as well as their actual orientation in biological assays and crystallographic experiments.

### 2.2. Chemistry

**Figure 4:**
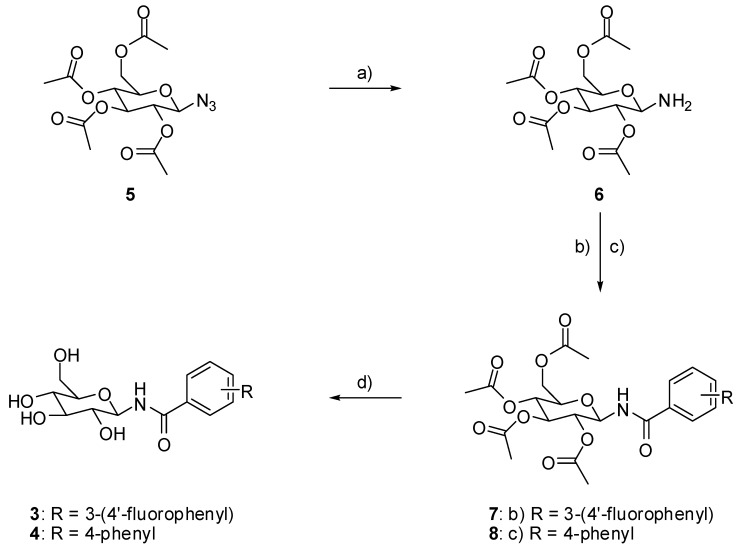
Synthesis of the designed compounds **3** and **4**. (**a**) Pd/C 10%, hydrogen, ethyl acetate, RT, 12 h, 97%; (**b**) 4’-fluorobiphenyl-3-carboxylic acid, COMU, DIPEA, DMF, 0 °C → RT, 12 h, 26%; (**c**) biphenyl-4-carbonylchloride, DIPEA, THF, RT, 2 h, 75%; (**d**) NaOMe, methanol, RT, 2 h, **3**: 53%, **4**: 73%.

Driven by our encouraging docking results we synthesized the designed molecules **3** and **4** according to the synthetic route summarized in Figure 4. The amino functionalized protected glucopyranoside derivative **6** could be obtained through hydrogenation of the commercially available azide **5**. The formation of an amide bond between the amine **6** and either 4’-fluorobiphenyl-3-carboxylic acid or biphenyl-4-carbonylchloride afforded the intermediates **7** and **8**. Elimination of the protecting acyl groups yielded the designed GP inhibitors **3** and **4**.

### 2.3. Biological Evaluation

The synthesized compounds were examined in in vitro assays to determine their inhibitory potential against GP. Inhibition constant (*K*_i_) values were measured using hlGPa (the pharmacologically relevant target) and the rabbit muscle enzymes (rmGPa and rmGPb) for comparison reasons since previously reported *K*_i_ values for other inhibitors were determined for the rabbit muscle enzymes. Production of rmGPa, rmGPb, and hlGPa, as well as enzyme kinetics experiments were performed according to previously described protocols [15]. As depicted in Table 1, both compounds **3** and **4** could be identified as GP inhibitors. They displayed competitive inhibition with respect to the substrate Glc-1-P (kinetics plots are presented in the Supplementary Materials, Figure S1). Within the series of tested molecules, **4** proved to be the most potent inhibitor with a *K*_i_ of 9.7 μM against rmGPb and 19.4 μM against the pharmacologically relevant hlGPa. Compared with the co-crystallized ligand **2** (PDB 3G2N), which was used for the design of the novel compounds, inhibitor **4** displayed elevated potency by almost one order of magnitude.

### 2.4. X-Ray Crystallography

Motivated by the positive results in the biological assays, we performed structural studies of the two novel inhibitors **3** and **4**. The studies were performed using rmGPb crystals since they are easier to grow and the active site is fully conserved in terms of amino acid sequence and structural architecture compared to hlGPa [17,18]. Previous studies revealed that structural data deriving from rmGPb can be directly correlated to hlGPa [15,19,20].

**Figure 5:**
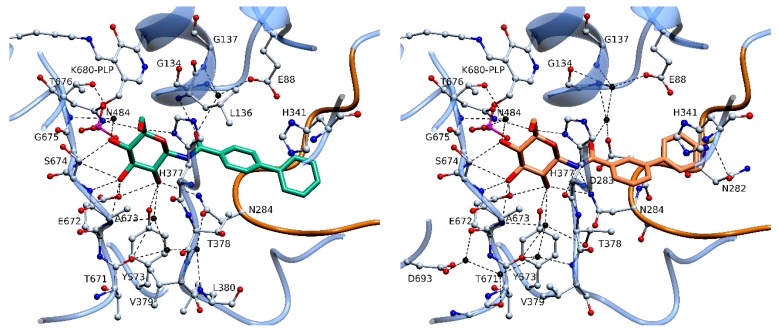
The binding mode of **4** (**left**) and **3** (**right**) at the active site of rmGPb. The inhibitor is shown in thick sticks, hydrogen bonds as dashed lines, and water molecules as black spheres.

Both inhibitors were found bound to the active site with the glucopyranose ring anchoring to the region known to bind glucose analogues in previous rmGPb-inhibitor complexes [2,21]. However, the two compounds bind significantly different at the enzyme active site and the biphenyl moieties point to different directions (Figure 5) as a result of their *meta* and *para* connection, respectively. As expected, the biphenyl fragment itself is not planar, and one benzene ring is twisted in relation to the other and the dihedral angle is 30° and 42° in **3** and **4**, respectively. Upon binding to rmGPb **3** and **4** engage in 16 and 14 hydrogen bond interactions with protein residues in the active site when applying a distance cut off of 3.3 Å using the program CONTACT as implemented in CCP4 [22] (Table S2), respectively. The –CONH- linker in the **3** complex forms two hydrogen bonds, the N1 to the carbonyl oxygen of His377 and to the side chain of Asn284, both of which are not present in the **4** complex. Instead, in the **4** complex, atom O2 of the linker forms a hydrogen bond to the main chain amide of Leu136 (Figure 5; Table S2), a hydrogen bond previously observed for other acyl-glucopyranose derivative complexes.

**Figure 6:**
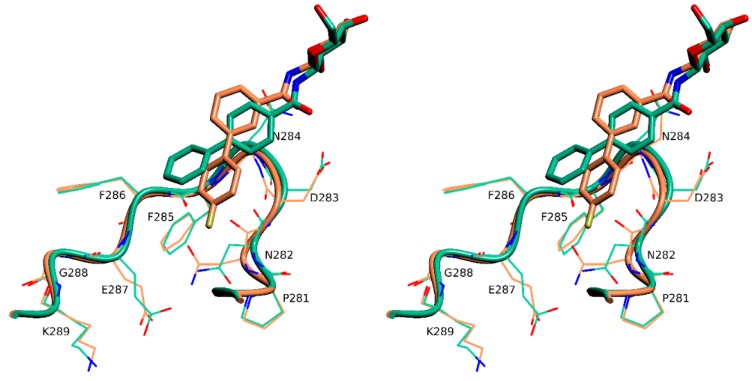
Stereo diagram of the superposition of the rmGPb-**3** complex (**brown**) onto the rmGPb-**4** complex (**green**) showing the different conformations of the 280s loop.

The fluorine of **3** forms a halogen [13] bond to the side chain of Asn282 (Figure 5) and this interaction seems to govern the orientation of the biphenyl moiety within the active site of rmGPb. Superposition of the two complex structures onto the native unliganded rmGPb structure reveals that the binding of **3** triggers a significant conformational change of a loop composed by residues 282–289 (termed 280s loop) [4]. The root mean square distance for all atoms of residues 282–285 between the rmGPb-**3** and rmGPb-**4** complex structures is 0.9 Å, with Asn282, Asp283, and Asn284, being the residues with the greatest difference. The new conformation of the 280s loop brings the side chain of Asn282 to a halogen bonding distance [13] to F1 of **3** (Figure 6). This conformation is further stabilized through the formation of a hydrogen bond between the main chain carbonyl oxygen of Asp283 and the main chain amide of Phe285.

## 3. Discussion

**Figure 7:**
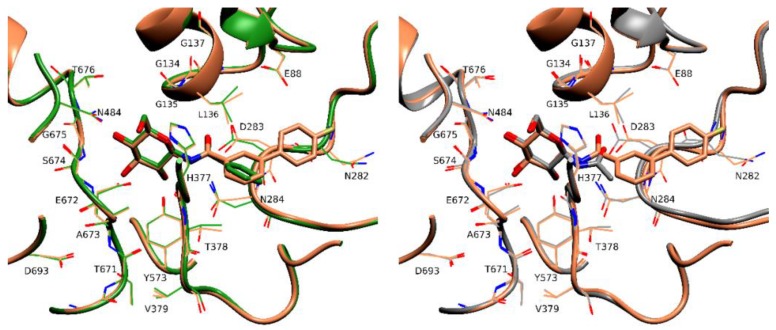
Superposition of the rmGPb-**3** complex (brown) onto the rmGPb-**2** (**left**, green)) and the rmGPb-**1** (**right**, grey) complexes.

Structural comparison of the rmGPb-**4** complex with the rmGPb-**2** complex [9] (Figure 7) reveals that although the glucopyranose atoms are at almost identical positions at the active site C1′ shifts by ~0.4 Å to best orient the biphenyl moiety (there is a 47° inclination between the phenyl rings of **2** and **4**) and hence the hydrogen bond of N1 to the main chain carbonyl oxygen of His377 is lost (distance in the rmGPb-**4** complex, 4.2 Å). However, this loss is compensated by the formation of a hydrogen bond of O2 to the main chain amide of Leu136. Therefore, it seems that the 15 additional van der Waals contacts (applying a distance cut off of 4.0 Å using the program CONTACT as implemented in CCP4 [22]) of the terminal phenyl group with the protein residues at the active site are the reason for the 8.3 times lower *K*_i_ value of **4** with respect to that of **2**. Similarly, structural comparison of the rmGPb-**4** complex and the rmGPb-**1** complex [16] (Figure 7) also reveals a C1′ shift by ~0.6 Å with a resulting loss of the hydrogen bond of N1 to the main chain carbonyl oxygen of His377 which is compensated by the formation of a hydrogen bond between O2 and Leu136. The van der Waals interactions of the biphenyl moiety once again seem to be the reason for the 3.3 times difference between the *K*_i_ values of **4** and **1**.

The biphenyl moiety in both inhibitors, **3** and **4**, is involved in 23 van der Waals interactions within a distance cut off of 4.0 Å with protein residues at the β-pocket, in each protein ligand complex. Therefore, it seems that despite their different location within the active site, non-polar interactions support the binding of the biphenyl moiety. Furthermore, despite the fact that the two terminal phenyl groups of **3** and **4** are inclined by ~15° to each other, both are involved in cation-π stacking interactions to the imidazole ring of His341 (Figure 5). The hydrogen bonds of N1 in the **4** complex to Asn284 and His377 and the halogen bond of F1 to Asn282 are partly counterbalanced in the **3** complex by the hydrogen bond of O2 to Leu136. Thus, the structural basis of the variation in the inhibitory potency between **3** and **4** seems to lie within the energy cost associated with the significant conformational change of the 280s loop triggered by the binding of **3** but not by the binding of **4**.

**Figure 8:**
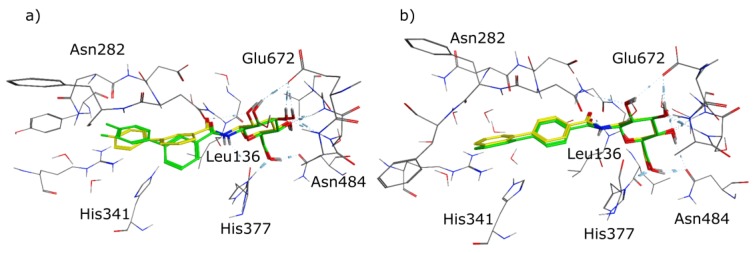
(**a**) Overlay of the second highest ranked docking pose of **3** in PDB 3G2N with carbon atoms in yellow, and the crystal structure of **3** in GP with carbon atoms in green, (**b**) overlay of the highest ranked docking pose of **4** in PDB 3G2N with carbon atoms in yellow, and the crystal structure of **4** in GP with carbon atoms in green.

The observed crystallographic data correspond very well with our design strategy and our molecular docking experiments. The second highest ranked docking pose of **3** is oriented like the co-crystallized ligand, pointing its *para* fluorinated phenyl ring towards the β-channel (Figure 8a). For compound **4** the congruence is even more striking as the docked pose and the observed orientation in the co-crystal structure are identical. Even the torsion between the two phenyl rings is modelled correctly (Figure 8b). The orientation of **3** with the *para* fluorinated aromatic ring in the β-channel offers a further potential explanation for its diminished inhibitory potency compared to **4**. Compared with the co-crystal structure PDB 3G2N, an ordered water molecule bound in this area by the formation of two hydrogen bonds with protein residues is displaced by **3**. As this area of the pocket is rather polar, it is plausible that the displacement of a water molecule by a hydrophobic system declines the constructive interactions and therefore worsens the inhibitors potency.

Given the unexpected significant difference in the potency of the two inhibitors, which can be explained by the X-ray crystallography results, the present study highlights the importance of obtaining structural data in every step of the inhibitor optimization process.

## 4. Materials and Methods

### 4.1. General

All NMR spectra were recorded on a Bruker (Fällanden, Switzerland) AVANCE III HD 500 One Bay spectrometer with a magnetic field of 11.75 T. For ^1^H NMR spectra a frequency of 500 MHz resulted. Chemical shifts are reported in ppm from tetramethylsilane as internal standard. Data are reported as follows: chemical shift, multiplicity (s = singlet, d = doublet, t = triplet, q = quartet, quint. = quintet, br. = broad, m = multiplet), coupling constants (Hz), integration. For the ^13^C NMR spectra a frequency of 125 MHz resulted. Chemical shifts are reported in ppm from tetramethylsilane as internal standard, whereas fluorine coupling was observed, it was reported with multiplicity (d = doublet), coupling constants (Hz), and number of carbon atoms. The multiplicities of the signals were determined by DEPT measurements. Low-resolution mass spectrometry was performed on a MSQ Plus device (Thermo Scientific, Basel, Switzerland). NMR spectra of all synthesized compounds as well as LC-MS and HPLC spectra of the test compounds, graphs of the kinetics studies and crystallographic data can be found in the Supplementary Materials.

### 4.2. Chemistry

All reagents and solvents were purchased from Sigma Aldrich (Buchs, Switzerland), TCI (Zwijndrecht, Belgium) or Fluorochem (Hadfield, UK) and used as received. Solvents were stored over 4 Å molecular sieves.

*2-(acetoxymethyl)-6-aminotetrahydro-2H-pyran-3,4,5-triyl triacetate* (**6**; ZHAWOC6075): 1-Azido-1-deoxy-b-D-glucopyranoside tetraacetate (**5**) (0.55 g, 1.78 mmol) was dissolved in ethyl acetate (20 mL). Palladium 10% on activated charcoal (0.11 g, 0.1 mmol) was added and a hydrogen atmosphere was applied at 1 bar. After stirring at ambient temperature for 12 h the mixture was filtered over celite and concentrated in vacuum. The title compound **6** (0.49 g, 97% yield) was used in further synthesis without purification: ^1^H-NMR (500 MHz, [D_6_]DMSO, 25 °C, TMS): δ = 5.23 (t, *J* = 9.6 Hz, 1H), 4.82 (t, *J* = 10.0 Hz, 1H), 4.62 (dd, *J* = 9.6 Hz, 9.0 Hz, 1H), 4.29 (d, *J* = 7.6 Hz, 1H), 4.09 (dd, *J* = 12.3 Hz, 5.3 Hz, 1H), 3.97 (dd, *J* = 12.3 Hz, 2.4 Hz, 1H), 3.88 (ddd, *J* = 10.0 Hz, 5.3 Hz, 2.4 Hz, 1H), 2.54 (br. s, 2H), 2.19 (s, 3H), 1.97 (s, 3H), 1.97 (s, 3H), 1.93 (s, 3H) ppm. ^13^C-NMR (125 MHz, [D_6_]DMSO, 25 °C, TMS): δ = 170.54, 170.01, 196.83, 169.79, 84.58, 73.39, 72.69, 71.61, 69.22, 62.82, 21.06, 21.03, 20.87, 20.80 ppm. MS (*m/z*): 284 [M + H]^+^.

*2-(acetoxymethyl)-6-(4’-fluorobiphenyl-3-ylcarboxamido)tetrahydro-2H-pyran-3,4,5-triyl triacetate* (**7**; ZHAWOC6074): The amine **6** (100 mg, 0.353 mmol) and 4’-fluorobiphenyl-3-carboxylic acid (77 mg, 0.353 mmol) were dissolved in dimethylformamide (6 mL) and cooled to 0 °C. COMU (151 mg, 0.353 mmol) was added followed by diisopropylethylamine (0.12 mL) and the mixture was stirred at 0 °C. The reaction was stirred overnight and allowed to reach ambient temperature. Ethyl acetate (25 mL) was added and the organic phase was washed with 1N HCl (2 × 10 mL), NaHCO_3_ 10% (2 × 10 mL) and brine (2 × 10 mL). The organic layer was dried over sodium sulphate and concentrated in vacuum. Purification by chromatography on silica gel (Gradient 0-100% ethyl acetate in cyclohexane) afforded the title compound **7** in 26% yield: ^1^H-NMR (500 MHz, CDCl_3_, 25 °C, TMS): δ = 7.99 (t, *J* = 1.7 Hz, 1H), 7.73–7.70 (m, 1H), 7.69-7.66 (m, 1H), 7.60–7.56 (m, 2H), 7.51 (t, *J* = 7.7 Hz, 1H), 7.18–7.13 (m, 2H), 5.47 (t, *J* = 9.2 Hz, 1H), 5.41 (t, *J* = 9.5 Hz, 1H), 5.13 (dd, *J* = 10.0 Hz, 9.5 Hz, 1H), 5.08 (t, *J* = 9.6 Hz, 1H), 4.35 (dd, *J* = 12.6 Hz, 4.4 Hz, 1H), 4.12 (dd, *J* = 12.6 Hz, 2.3 Hz, 1H), 3.93 (ddd, *J* = 10.0 Hz, 4.4 Hz, 2.3 Hz, 1H), 2.08 (s, 3H), 2.05 (s, 3H), 2.05 (s, 3H), 2.05 (s, 3H) ppm. ^13^C-NMR (125 MHz, CDCl_3_, 25 °C, TMS): δ = 171.61, 170.63, 169.88, 169.61, 167.04, 162.79 (d, *J* = 247.4 Hz, 1C), 140.89, 136.01 (d, *J* = 3.3 Hz, 1C), 133.42, 130.84, 129.29, 128.76 (d, *J* = 8.2, 2C), 126.12, 125.62, 115.88 (d, *J* = 21.5 Hz, 2C), 78.99, 73.66, 72.58, 70.90, 68.22, 61.64, 20.76, 20.74, 20.62, 20.61 ppm. MS (*m/z*): 546 [M + H]^+^.

*2-(acetoxymethyl)-6-biphenyl-4-ylcarboxamidotetrahydro-2H-pyran-3,4,5-triyl triacetate* (**8**; ZHAWOC6076): Under an argon atmosphere **6** (50 mg, 0.176 mmol) was dissolved in tetrahydrofuran (3 mL). Biphenyl-4-carbonylchloride (38 mg, 0.176 mmol) and diisopropylethylamine (0.05 mL) were added and the mixture was stirred at ambient temperature for 2 h. The solvent was removed in vacuum and the crude material was purified by chromatography on silica gel (Gradient: 0–100% methanol in dichloromethane) to obtain the title compound **8** in 75% yield: ^1^H-NMR (500 MHz, CDCl_3_, 25 °C, TMS): δ = 7.84–7.83 (m, 2H), 7.70–7.66 (m, 2H), 7.64–7.60 (m, 2H), 7.50–7.45 (m,2H), 7.43–7.38 (m, 1H), 7.15 (d, *J* = 9.1 Hz, 1H), 5.49 (t, *J* = 9.3 Hz, 1H), 5.49 (t, *J* = 9.5 Hz, 1H), 5.14 (t, *J* = 10.0, 1H), 5.10 (t, *J* = 9.5 Hz, 1H), 4.37 (dd, *J* = 12.7 Hz, 4.4 Hz, 1H), 4.13 (dd, *J* = 12.7 Hz, 2.2 Hz, 1H), 3.94 (ddd, *J* = 10.0 Hz, 4.4 Hz, 2.2 Hz, 1H), 2.10 (s, 3H), 2.07 (s, 9H) ppm. ^13^C-NMR (125 MHz, CDCl_3_, 25 °C, TMS): δ = 171.57, 170.63, 169.88, 169.61, 166.85, 145.19, 139.73, 131.35, 128.96, 128.19, 127.80, 127.38, 127.21, 78.97, 73.63, 72.63, 70.86, 68.25, 61.66, 20.75, 20.74, 20.62, 20.61 ppm. MS (*m/z*): 528 [M + H]^+^.

*4’-fluoro-N-(3,4,5-trihydroxy-6-(hydroxymethyl)tetrahydro-2H-pyran-2-yl)biphenyl-3-carboxamide* (**3**; ZHAWOC6072): The acyl protected compound **7** (50 mg, 0.10 mmol) was dissolved in methanol (15 mL) and sodium methanolate (54 mg, 1.00 mmol in 1 mL methanol) was added and the mixture kept stirring at ambient temperature for 2h. After neutralization with Amberlyst 15 H^+^ form and further stirring for 5 min. the mixture was filtrated and the solvent was removed in vacuum. Purification by chromatography on reversed phase silica gel (Gradient 0–100% methanol in water) afforded the title compound **3** as a white solid with purity > 99.8% (0.02 g, 53% yield): ^1^H-NMR (500 MHz, [D_6_]DMSO, 25 °C, TMS): δ = 8.99 (d, *J* = 9.0 Hz, 1H), 8.19 (t, *J* = 1.6 Hz, 1H), 7.89 (dt, *J* = 7.8 Hz, 1.1 Hz, 1H), 7.85–7.79 (m, 3H), 7.58–7.54 (m, 1H), 7.37–7.30 (m, 2H), 5.05–4.89 (m, 4H), 4.52 (t, *J* = 5.6Hz, 1H), 3.72–3.65 (m, 1H), 3.48–3.41 (m, 1H), 3.37–3.31 (m, 1H), 3.25 (td, *J* = 8.8 Hz, 4.2 Hz, 1H), 3.20 (ddd, *J* = 9.4 Hz, 5.6 Hz, 2.0 Hz, 1H), 3.11 (td, *J* = 9.3 Hz, 4.8 Hz, 1H) ppm. ^13^C-NMR (125 MHz, [D_6_]DMSO, 25 °C, TMS): δ = 166.88, 162.53 (d, *J* = 244.8 Hz, 1C), 139.48, 136.45 (d, *J* = 3.2 Hz, 1C), 135.27, 130.04, 129.47, 129.39 (d, *J* = 8.2 Hz, 2C), 127.33, 126.06, 116.24, (d, *J* = 21.4 Hz, 2C), 80.79, 79.26, 77.99, 72.67, 70.52, 61.47 ppm. MS (*m/z*): 378 [M + H]^+^.

*N-(3,4,5-trihydroxy-6-(hydroxymethyl)tetrahydro-2H-pyran-2-yl)biphenyl-4-carboxamide* (**4**; ZHAWOC6077): The acyl protected compound **8** (70 mg, 0.133 mmol) was dissolved in methanol (15 mL) and sodium methanolate (72 mg, 1.33 mmol in 1 mL methanol) was added and the mixture kept stirring at ambient temperature for 2 h. After neutralization with Amberlyst 15 H^+^ form and further stirring for 5 min. the mixture was filtrated and the solvent was removed in vacuum. Purification by chromatography on reversed phase silica gel (gradient 0–100% methanol in water) afforded the title compound **4** as a white solid with purity >99.8% (0.04 g, 73% yield): ^1^H-NMR (500 MHz, [D_6_]DMSO, 25 °C, TMS): δ = 8.89 (d, *J* = 8.9 Hz, 1H), 8.04–8.00 (m, 2H), 7.81–7.77 (m, 2H), 7.76–7.72 (m, 2H), 7.52–7.47 (m, 2H), 7.44–7.39 (m, 1H), 5.04–4.89 (m, 4H), 4.52 (t, *J* = 5.7 Hz, 1H), 3.69 (m, 1H), 3.45 (dt, *J* = 11.5 Hz, 5.6 Hz, 1H), 3.36 (td, *J* = 9.0 Hz, 4.7 Hz, 1H), 3.25 (td, *J* = 8.8 Hz, 4.4 Hz, 1H), 3.19 (ddd, *J* = 9.7 Hz, 5.6 Hz, 2.1 Hz, 1H), 3.11 (td, *J* = 9.2 Hz, 5.0 Hz, 1H) ppm. ^13^C-NMR (125 MHz, [D_6_]DMSO, 25 °C, TMS): δ = 166.74, 143.40, 139.58, 133.41, 129.51, 128.79, 128.57, 127.36, 126.86, 80.81, 79.26, 78.08, 72.56, 70.55, 61.48 ppm. MS (*m/z*): 360 [M + H]^+^.

### 4.3. In Silico Studies

Molecular modelling experiments were performed using the Molecular Operating Environment MOE 2015.10 from Chemical Computing Group. Co-crystal structures of GP are available from the Protein Data Bank. For the actual work pdb entry: 3G2N was selected for the computational studies. In MOE the pocket was prepared for the dockings via the Protonate 3D method applying the default values for temperature 300 K, pH 7 and salt 0.1. The ligands to be docked to the protein were imported from SD files to receive a MOE compatible molecular database. As the SD files did not contain 3D coordinates, they were generated directly using MOE rebuild3D with an RMSD gradient of 0.1. For docking experiments the Amber10:EHT force field was used [23,24]. The pharmacophore placement was applied with a rigid receptor. The docked poses were subsequently analysed with respect to their scores and interactions with the target enzyme.

### 4.4. Kinetics

Rabbit muscle GPb (rmGPb) was purified from rabbit skeletal muscles following the protocol developed by Fischer and Krebs [25] with a slight modification (l-cysteine was replaced with 2-mercaptoethanol). Human liver GPb (hlGPb) was produced as described previously [15]. rmGPa and hlGPa were prepared by phosphorylation of rmGPb and hlGPb, respectively, performed using a truncated form of the γ (catalytic) subunit of rabbit skeletal muscle phosphorylase kinase produced as described previously [26].

Kinetic studies were performed at 30 °C in the direction of glycogen synthesis by measuring the inorganic phosphate released in the reaction using the method by Saheki et al. [27] 3 µg/mL rmGPb, rmGPa, or 1 µg/mL hlGPa were assayed in a 30 mM imidazole/HCl buffer (pH 6.8) containing 60 mM KCl, 0.6 mM EDTA, and 0.6 mM dithiothreitol using constant concentrations of glycogen (0.2% *w/v*), AMP (1 mM; only for the rmGPb experiments), and various concentrations of Glc-1-P (2, 3, 4, 6, and 10 mM for rmGP and 1, 2, 3, 4, and 6 mM for hlGPa) and inhibitors. Briefly, absorption at 850 nm of each sample is transformed to µmoles of phosphates by using a standard curve. Initial velocities were calculated from the pseudo-first order rate constants (*k*) using the first-order rate equation ([A]=[A]_0_ ∙ e*^k^*^t^) where [A]_0_ and [A] are the initial and the sample’s concentration of substrate at various times, and *t* is the corresponding time (min). The apparent *K_M_* values (*K*_M(app.)_) are then calculated by plotting pseudo-first order rate constants (*k*) vs. [Glc-1-P] using the Michaelis–Menten equation. The inhibition constant (*K*_i_) values were then calculated from the intercept to horizontal axis of the plot of *K*_M(app.)_ vs. [inhibitor] using non-linear regression program GRAFIT [28] and an explicit value for the standard deviation of each point.

### 4.5. X-Ray Crystallography

Tetragonal (space group *P*4_3_2_1_2) T state rmGPb crystals were grown by the batch method. Briefly, an rmGPb (100 mg/mL) solution in a 50 mM β-glycerol phosphate buffer pH 6.8, supplemented with 50 mM β-mercaptoethanol and 1 mM EDTA was dialyzed overnight at 4 °C against a solution of a 10 mM BES (*N*,*N*-bis-(2-hydroxyethyl)-2-aminoethane sulfonic acid/NaOH) buffer pH 6.7, supplemented with 0.1 mM EDTA, 0.02% (*w/v*) sodium azide and active charcoal (rmGPb—charcoal ratio 1:1.2) to remove any nucleotides bound to the enzyme. The enzyme solution was then diluted to 25–30 mg/mL with the dialysis buffer and the addition of spermine and DTT to final concentration of 1 and 3 mM, respectively. Microseeds, prepared from previously grown rmGPb crystals, were also added in the crystallization solution. The final crystallization solution was placed in small tubes (diameter 3 mm; length 3 cm) and left at 16 °C. rmGPb crystals appeared after 3–4 days. X-ray crystallographic binding studies were performed by diffusion of either **3** or **4** (1 mM; 24 h), solution in the crystallization media supplemented with 10% (*v/v*) DMSO in preformed rmGPb crystals at room temperature prior to data collection. X-ray diffraction data were collected using a Cu X-ray microfocus source (Oxford Diffraction SuperNova) equipped with a 4-kappa goniometer and the ATLAS CCD (135 mm) detector at room temperature. Crystal orientation, integration of reflections, inter-frame scaling, partial reflection summation, and data reduction was performed by the program CrysalisPro (Agilent Technologies UK Ltd.) [29]. Scaling and merging of intensities were performed by Aimless [30] and the optimum resolution was selected based on the *CC_1/2_* criterion [30]. Crystallographic refinement of the complexes was performed by maximum-likelihood methods using REFMAC [31]. The starting model employed for the refinement of the complexes was the structure of the native T state rmGPb complex determined at 1.9 Å resolution (Leonidas et al., unpublished results). Ligand molecule coordinates and topologies were constructed using AceDRG [32] within *Coot* [33] and they were fitted to the electron density maps after adjustment of their torsion angles. A summary of the data processing and refinement statistics for the inhibitor complex structures is given in Table S1 in the Supplementary Materials. The validity of the refinement procedure was checked using the PDB_REDO server [34]. As there were more than five reflections per atom available, both an isotropic and an anisotropic B-factor model were considered, and the isotropic B-factor model was selected based on the Hamilton R ratio test. A TLS model for grouped atom movement with one TLS group was used. The stereochemistry of the protein residues was validated by MolProbity [35]. Figures were prepared with CCP4 Molecular Graphics [36]. The coordinates of the new structures have been deposited with the RCSB Protein Data Bank (http://www.rcsb.org/pdb) with codes presented in Table S1.

## 5. Conclusions

Two novel inhibitors of GP were structure-based designed in silico, synthesized and evaluated towards their inhibitory properties from a kinetic and crystallographic point of view. Both inhibitors displayed higher potency towards rmGPb compared to the co-crystallized template ligand that was employed as the starting point for our targeted design. With a *K*_i_ of 9.7 μM for the rabbit muscle enzyme the most potent inhibitor was almost one order of magnitude more potent than the template compound. The pathologically relevant human target enzyme hlGPa was inhibited with a *K*_i_ of 19.4 μM. Crystallographic studies of the two inhibitors revealed that they bind as expected to the enzyme with the glucopyranose moiety anchored by the formation of multiple hydrogen bonds. Nevertheless, deviations were observed for the enzyme structure in the 280 s loop upon binding the two inhibitors. The resulting differences in constructive interactions to the inhibitor serve as the basis to explain the difference in potency of the two inhibitors. Comparison of the computationally derived ligand poses with the effectively determined coordinates obtained by X-ray crystallography demonstrated very high similarity, confirming the validity of the in silico drug design strategy.

## Figures and Tables

**Table 1:** Inhibitory data for glycogen phosphorylases.

Inhibitor	Structure	*K*_i_ [μM]		
		hlGPa	rmGPa	rmGPb
**1**	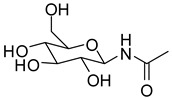	n.r. ^1^	35 ± 1 [16]	32 ± 1 [16]
**2**	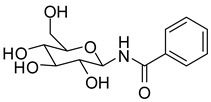	n.r. ^1^	n.r. ^1^	81 ± 7 [7]
**3** ^2^	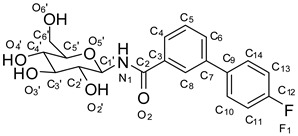	45.6 ± 1.8	31.4 ± 2.2	56.1 ± 4.4
**4** ^2^	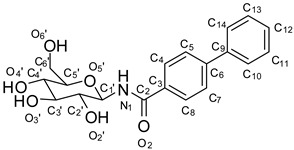	19.4 ± 0.2	12.8 ± 0.6	9.7 ± 0.7

^1^ Not reported; ^2^ crystallographic numbering.

## Supplementary Materials

Supplementary materials related to this article, including complete analytical data of the synthesized compounds, kinetics plots and crystallographic data, are available online.
