# Supplementary material for: High Consistency of Structure-Based Design and X-Ray Crystallography: Design, Synthesis, Kinetic Evaluation and Crystallographic Binding Mode Determination of Biphenyl-N-acyl-β-d-Glucopyranosylamines as Glycogen Phosphorylase Inhibitors
Source: Molecules. 2019 Apr 3;24(7):1322. doi: 10.3390/molecules24071322 (PMC6479789; doi:10.3390/molecules24071322)
Supplement: Supplementary file 1 [file molecules-24-01322-s001.zip › sup/final Supporting_Information_Molecules_revised.docx]

**Supplementary Materials**

**High consistency of structure-based design and X-ray crystallography: Design, synthesis, kinetic evaluation and crystallographic binding mode determination of biphenyl-N-acyl-β-D-glucopyranosylamines as glycogen phosphorylase inhibitors**

Thomas Fischer, Symeon M. Koulas, Anastasia S. Tsagkarakou, Efthimios Kyriakis, Vassiliki T. Skamnaki, Panagiota G.V. Liggiri, Spyros E. Zographos, Rainer Riedl* and Demetres D. Leonidas*

*2-(acetoxymethyl)-6-aminotetrahydro-2H-pyran-3,4,5-triyl triacetate* (**6**; *ZHAWOC6075*)

NMR


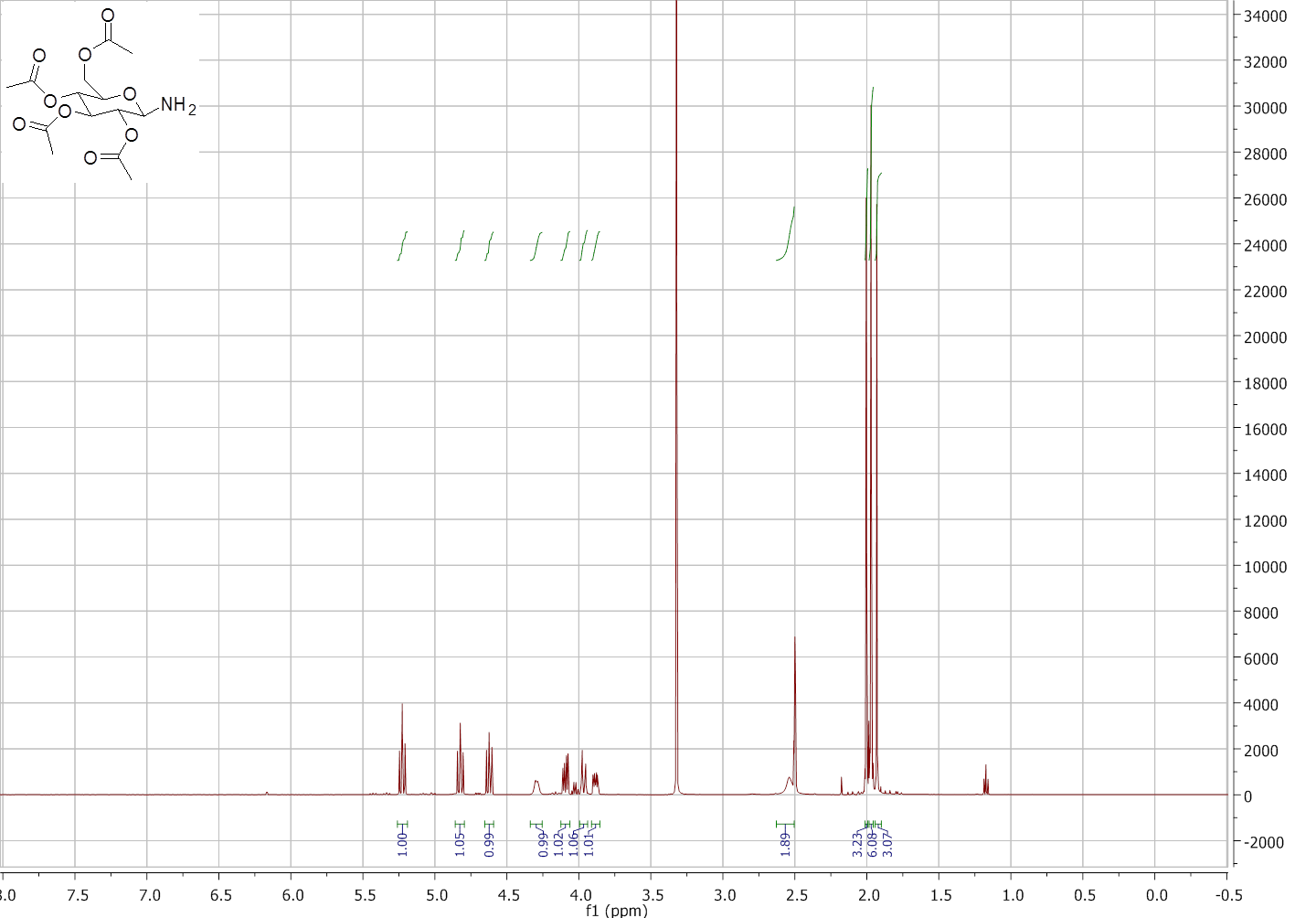


2-(acetoxymethyl)-6-aminotetrahydro-2H-pyran-3,4,5-triyl triacetate (**6**; ZHAWOC6075)


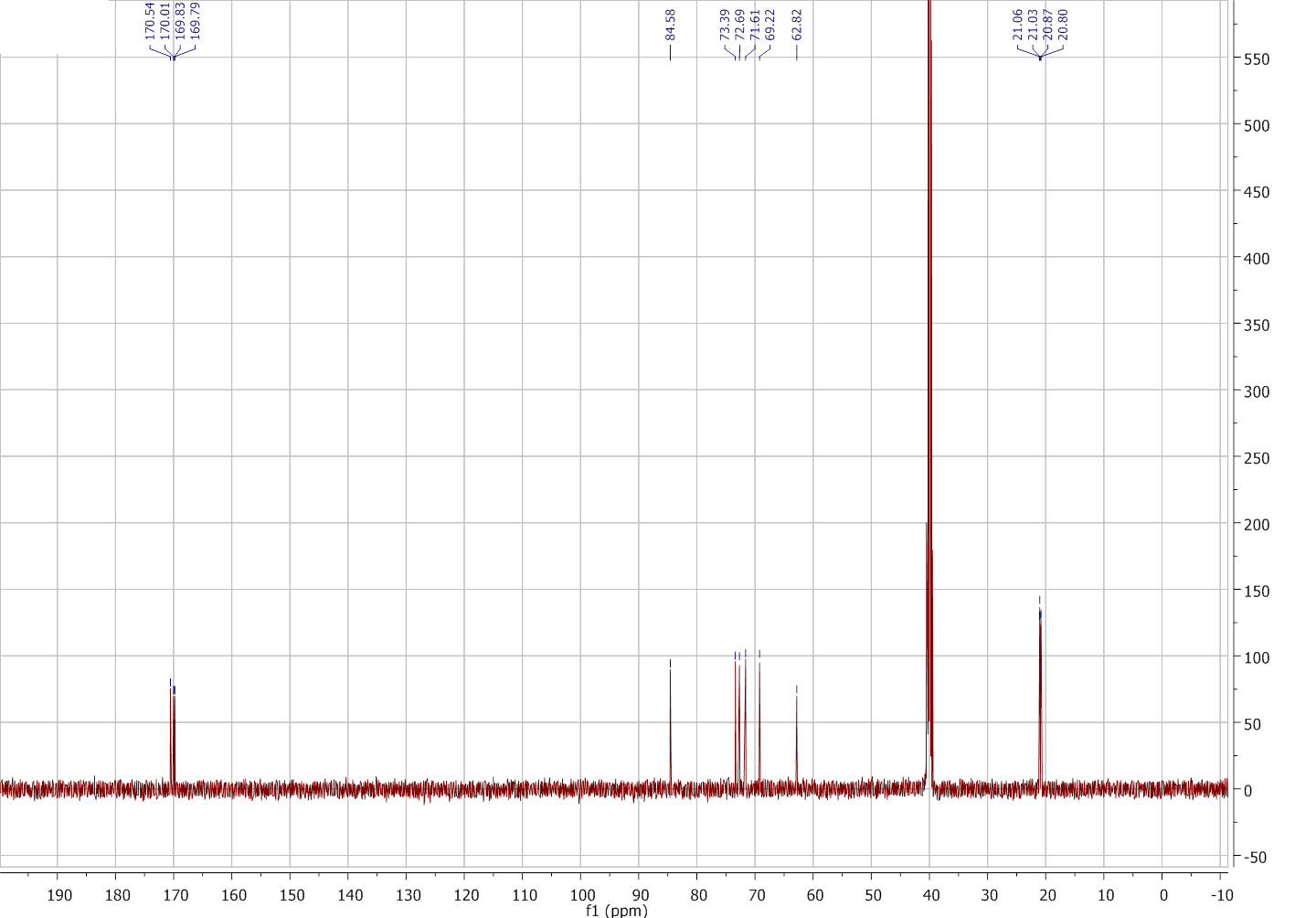


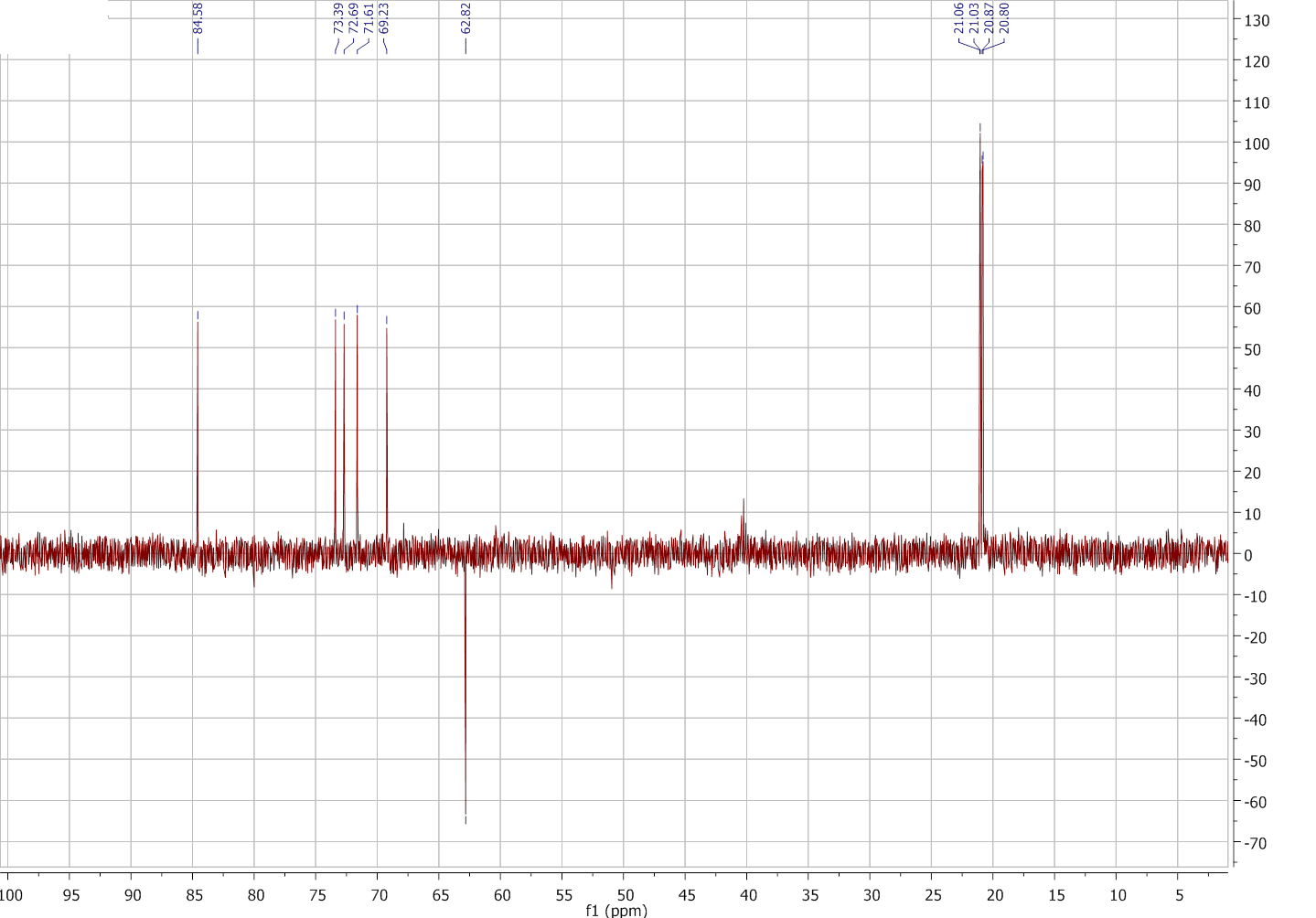


2-(acetoxymethyl)-6-(4'-fluorobiphenyl-3-ylcarboxamido)tetrahydro-2H-pyran-3,4,5-triyl triacetate (**7**; ZHAWOC6074)

NMR


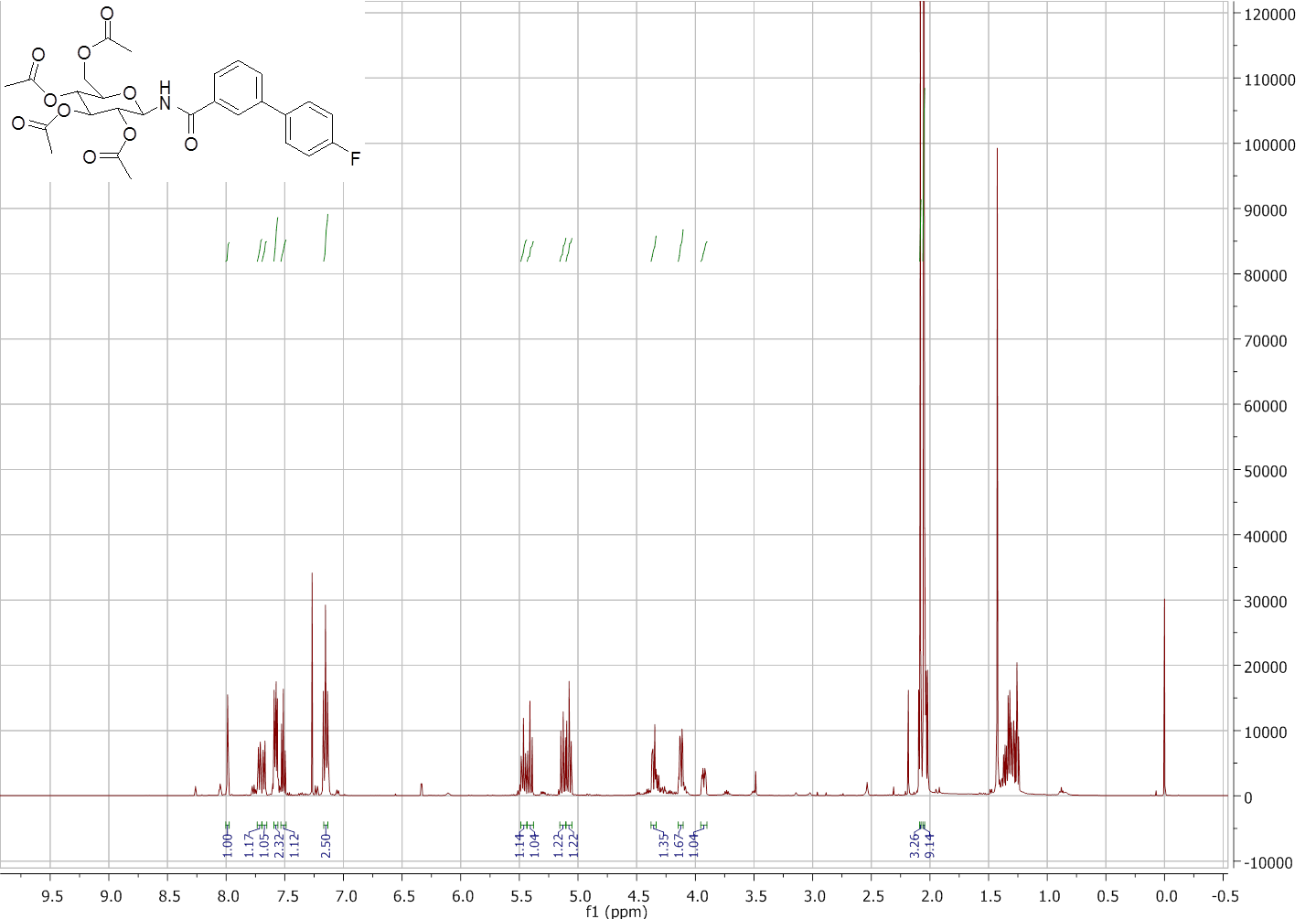


2-(acetoxymethyl)-6-(4'-fluorobiphenyl-3-ylcarboxamido)tetrahydro-2H-pyran-3,4,5-triyl triacetate (**7**; ZHAWOC6074)


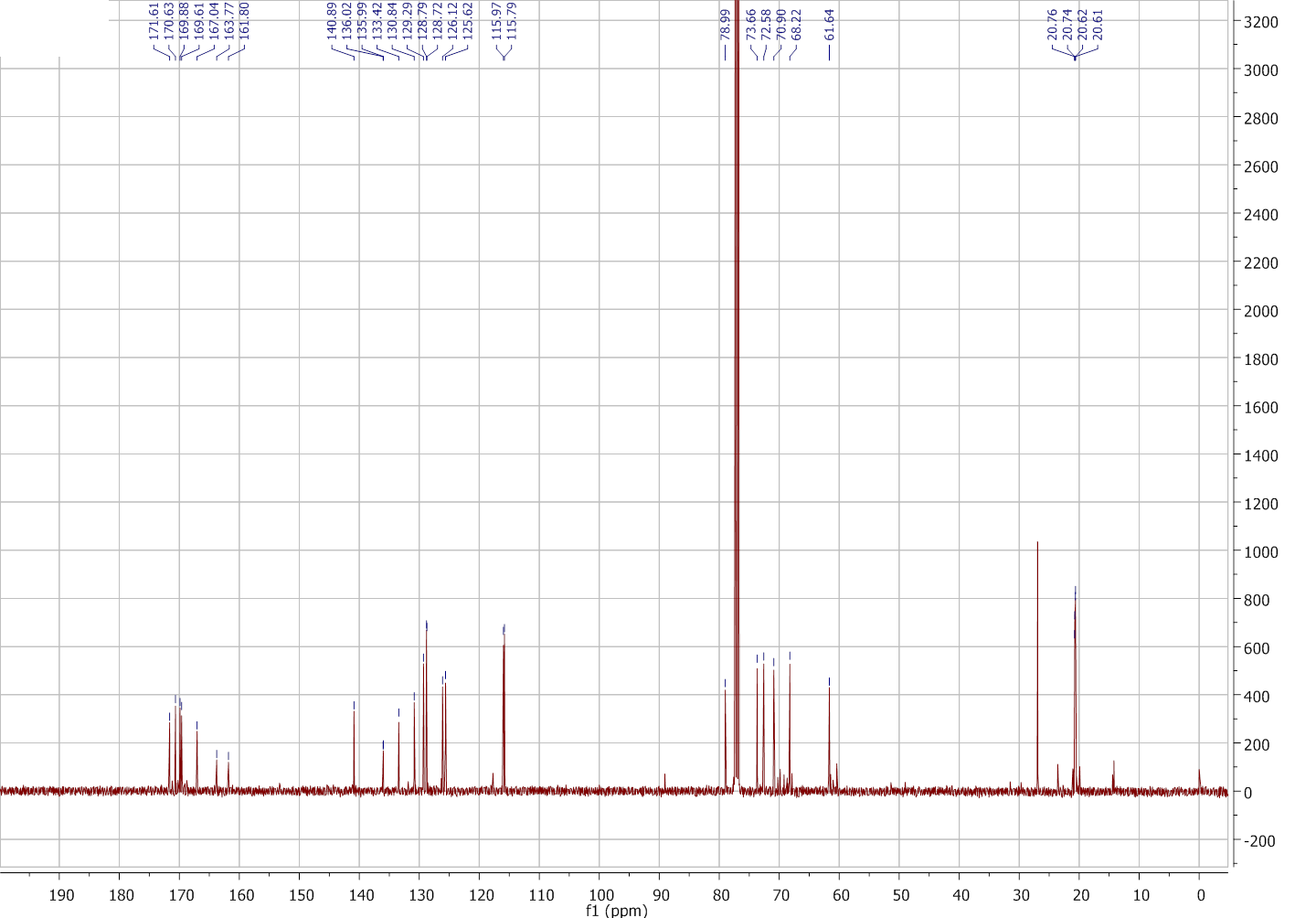


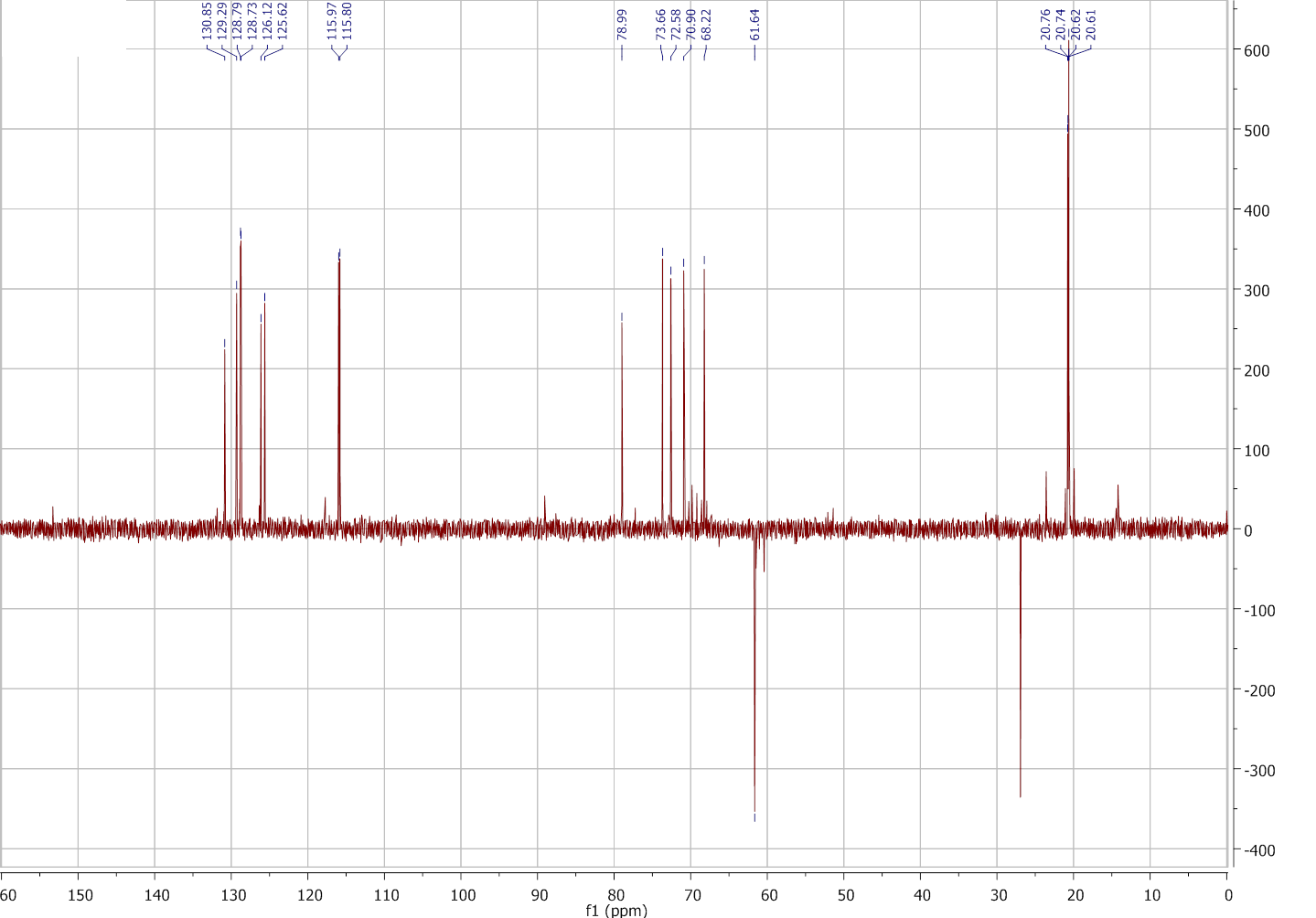


2-(acetoxymethyl)-6-biphenyl-4-ylcarboxamidotetrahydro-2H-pyran-3,4,5-triyl triacetate (**8**; ZHAWOC6076)

NMR


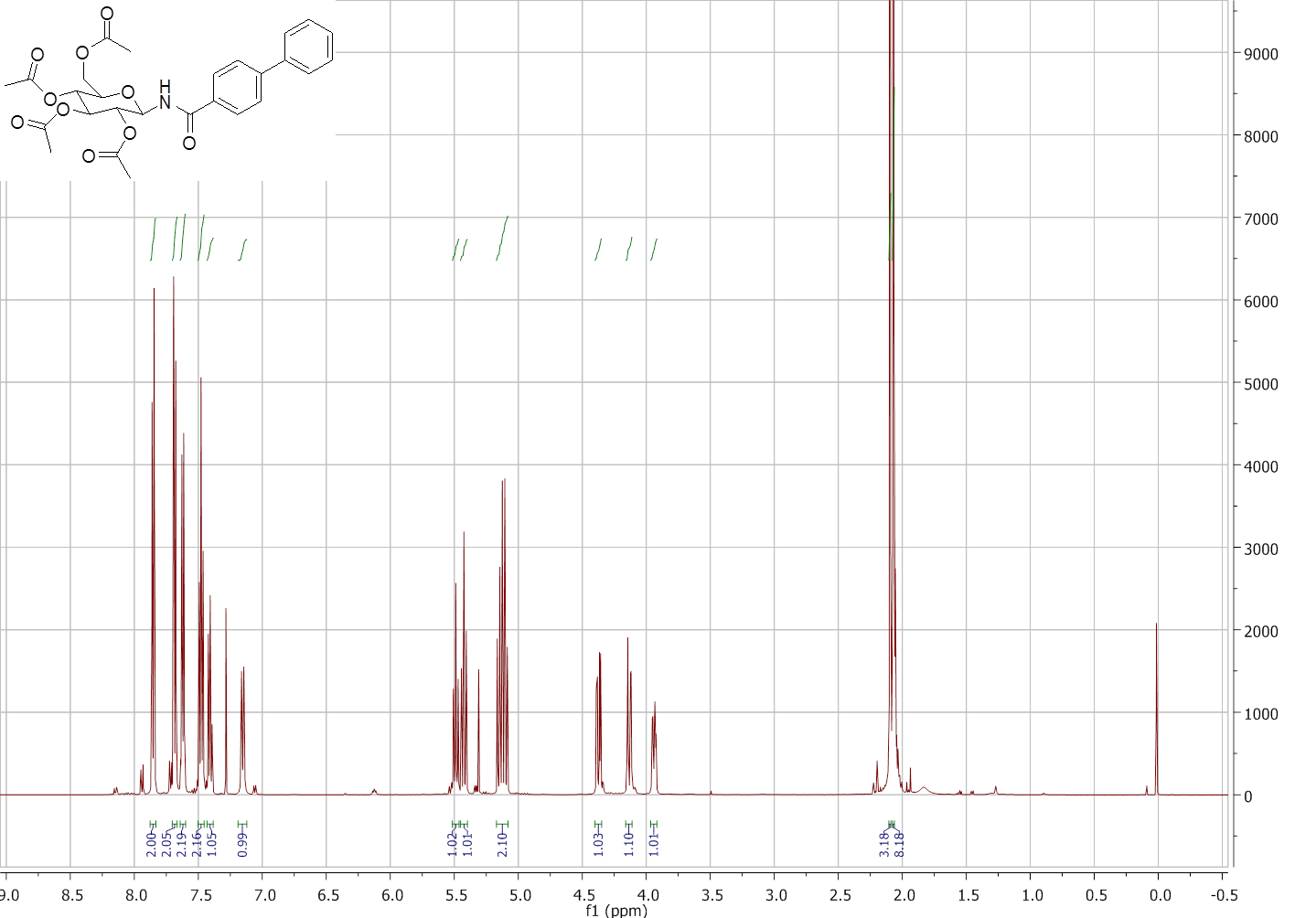


2-(acetoxymethyl)-6-biphenyl-4-ylcarboxamidotetrahydro-2H-pyran-3,4,5-triyl triacetate (**8**; ZHAWOC6076)


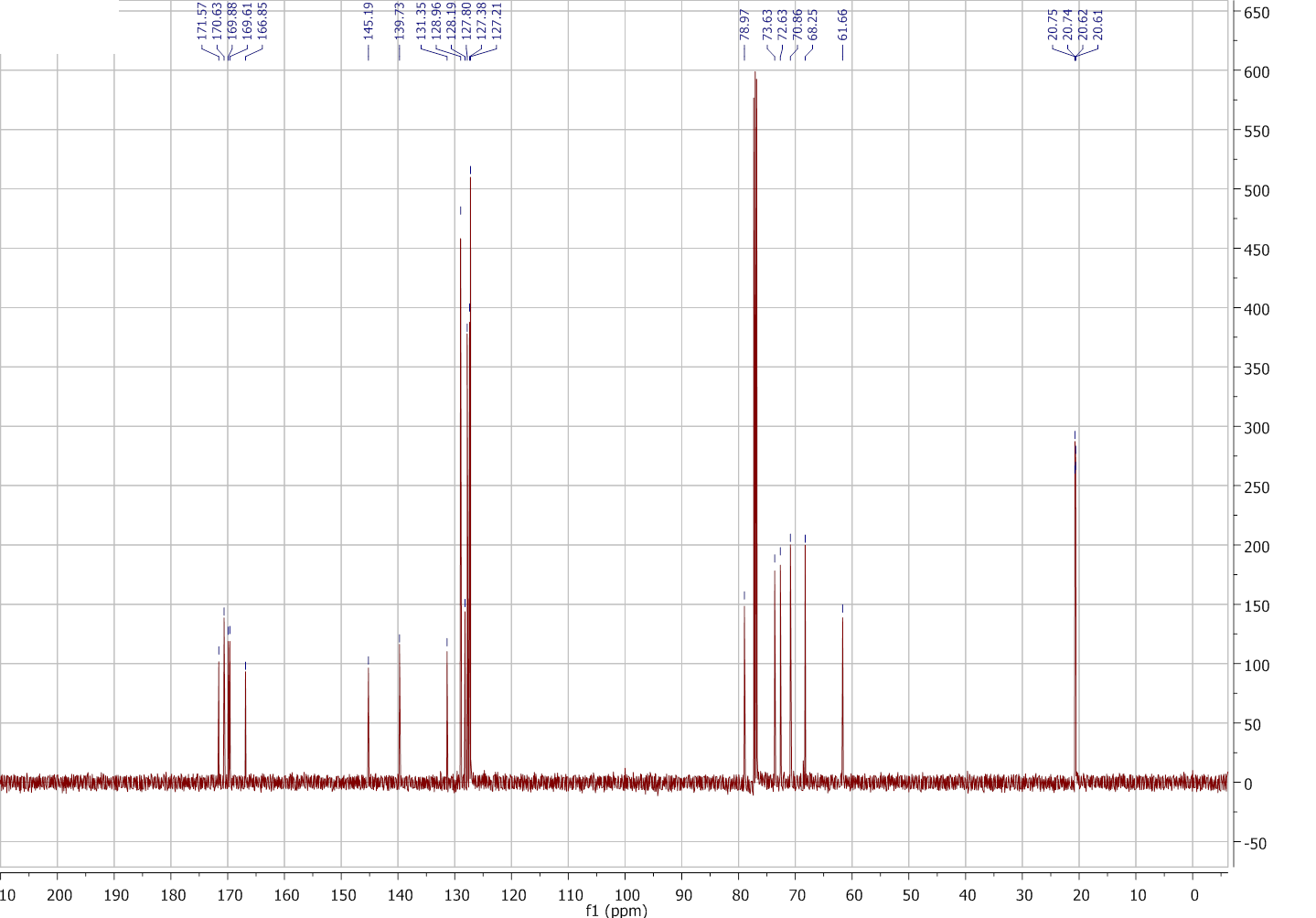


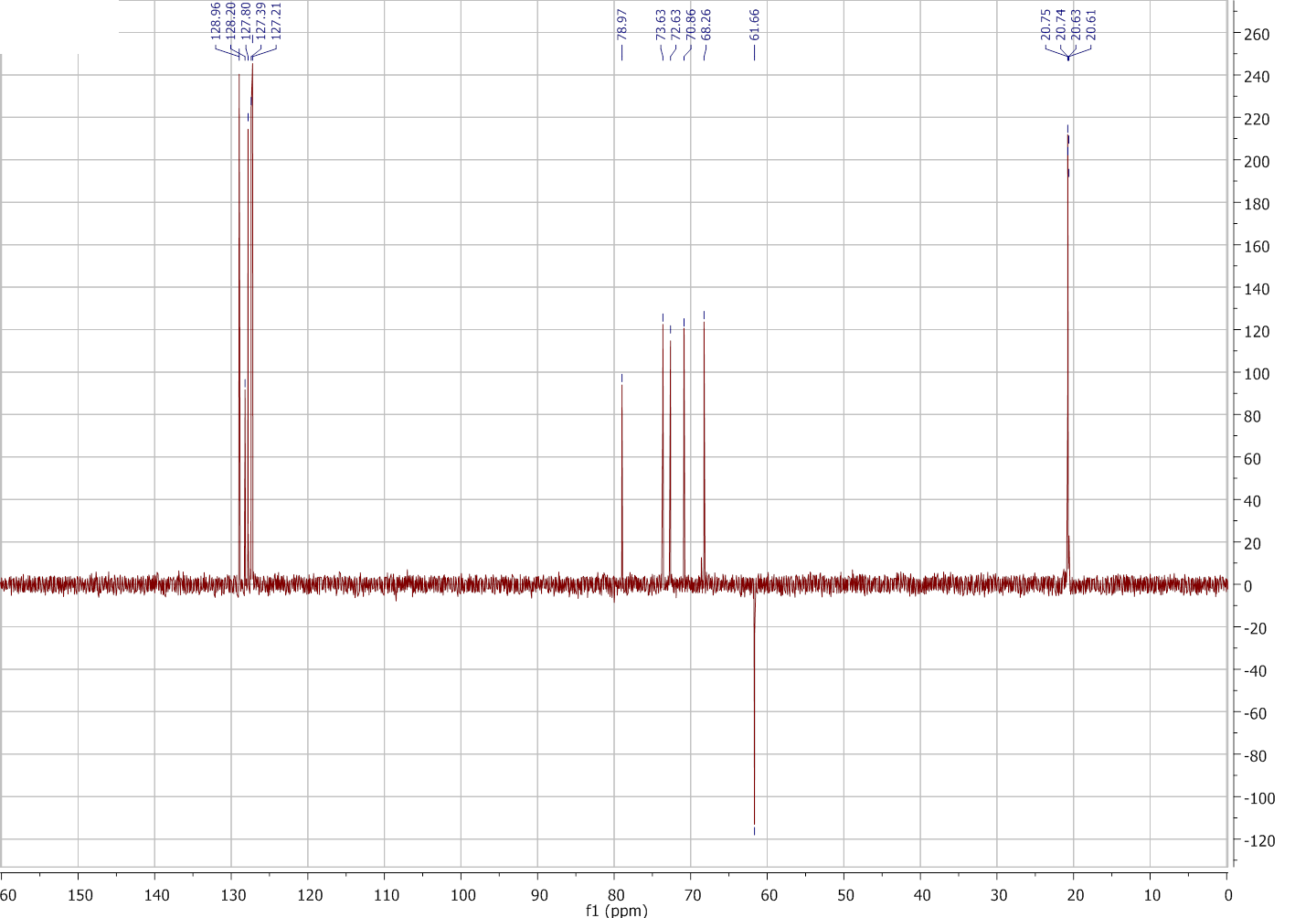


4'-fluoro-N-(3,4,5-trihydroxy-6-(hydroxymethyl)tetrahydro-2H-pyran-2-yl)biphenyl-3-carboxamide (**3**; ZHAWOC6072)

NMR


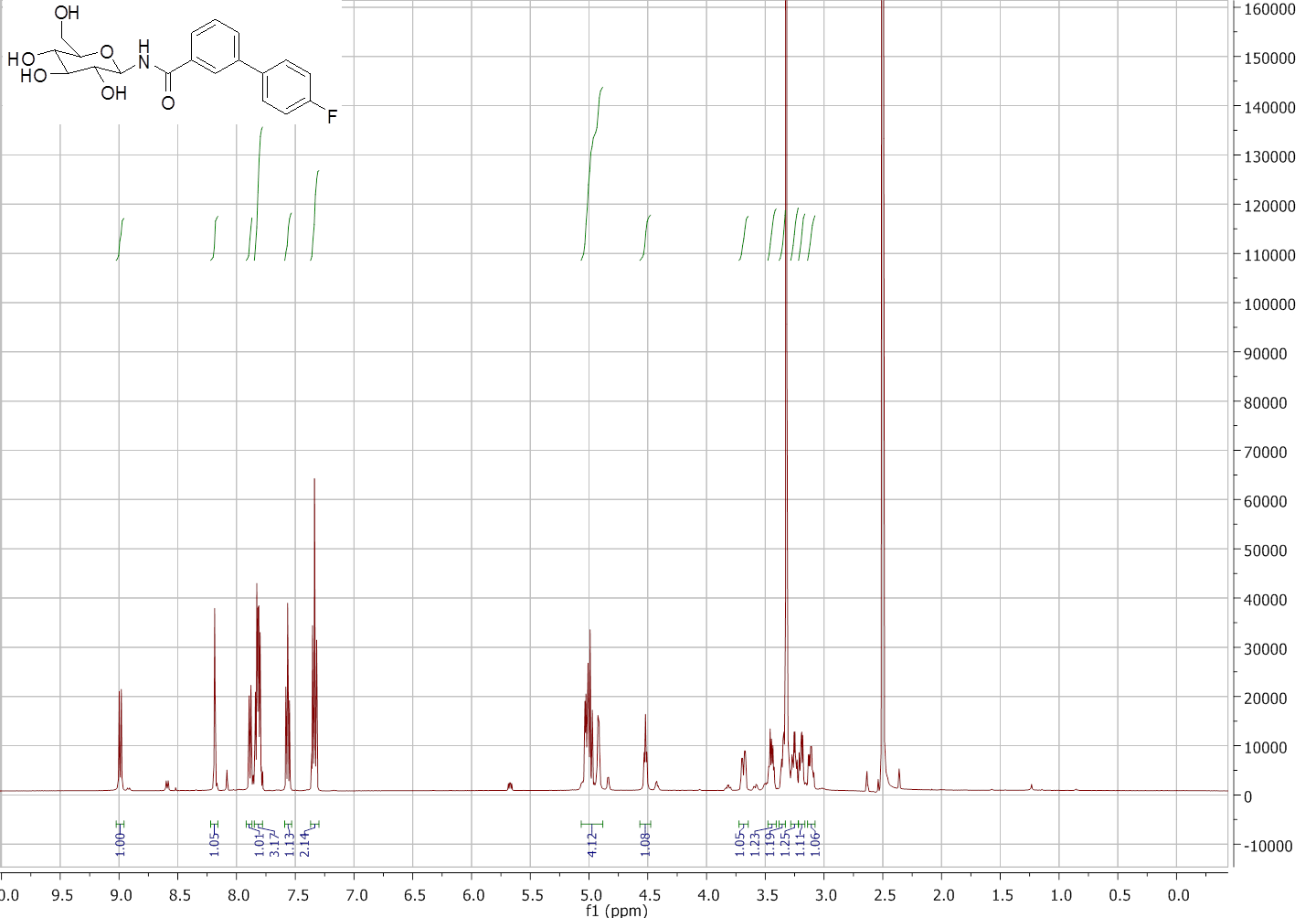


4'-fluoro-N-(3,4,5-trihydroxy-6-(hydroxymethyl)tetrahydro-2H-pyran-2-yl)biphenyl-3-carboxamide (**3**; ZHAWOC6072)


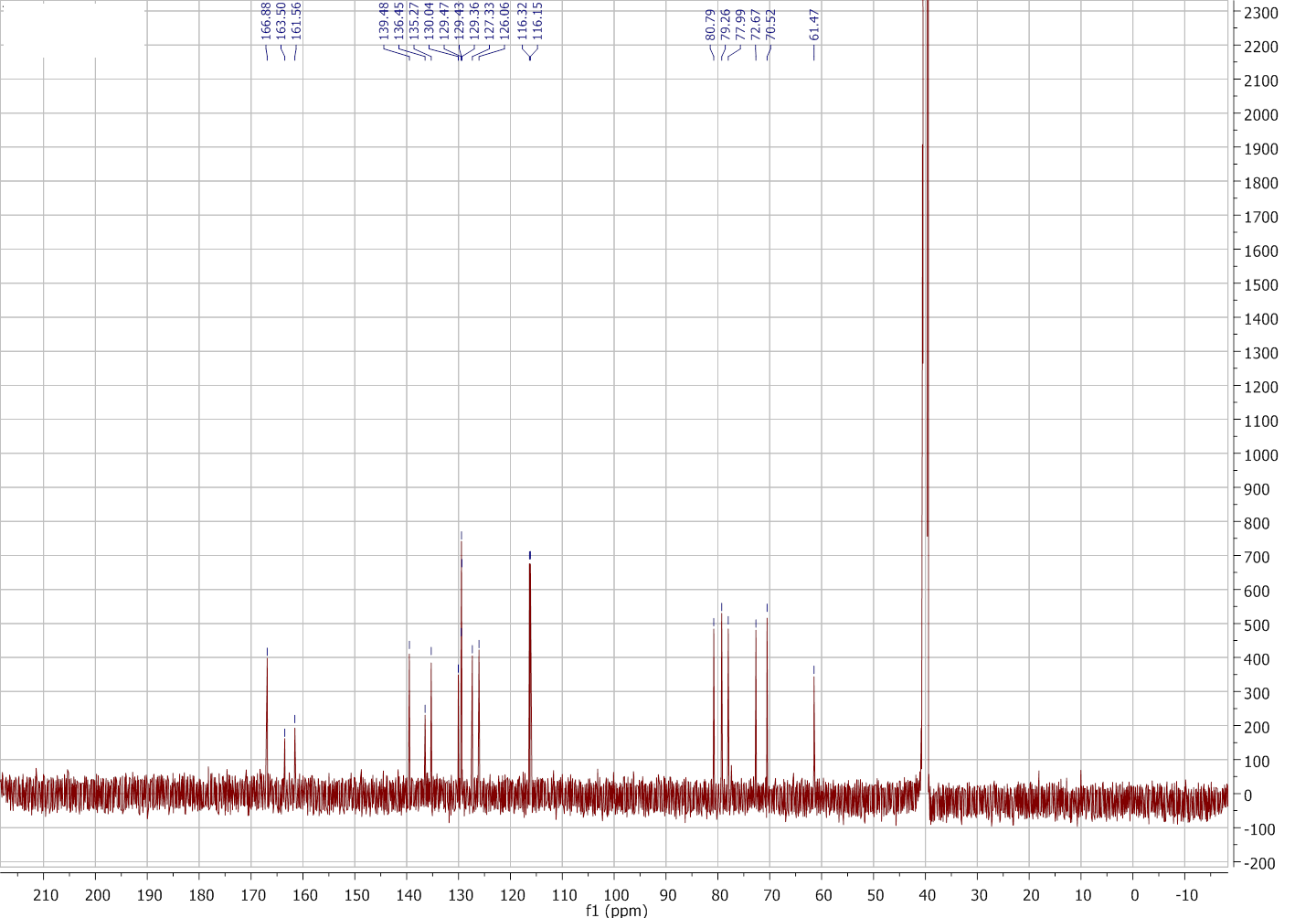


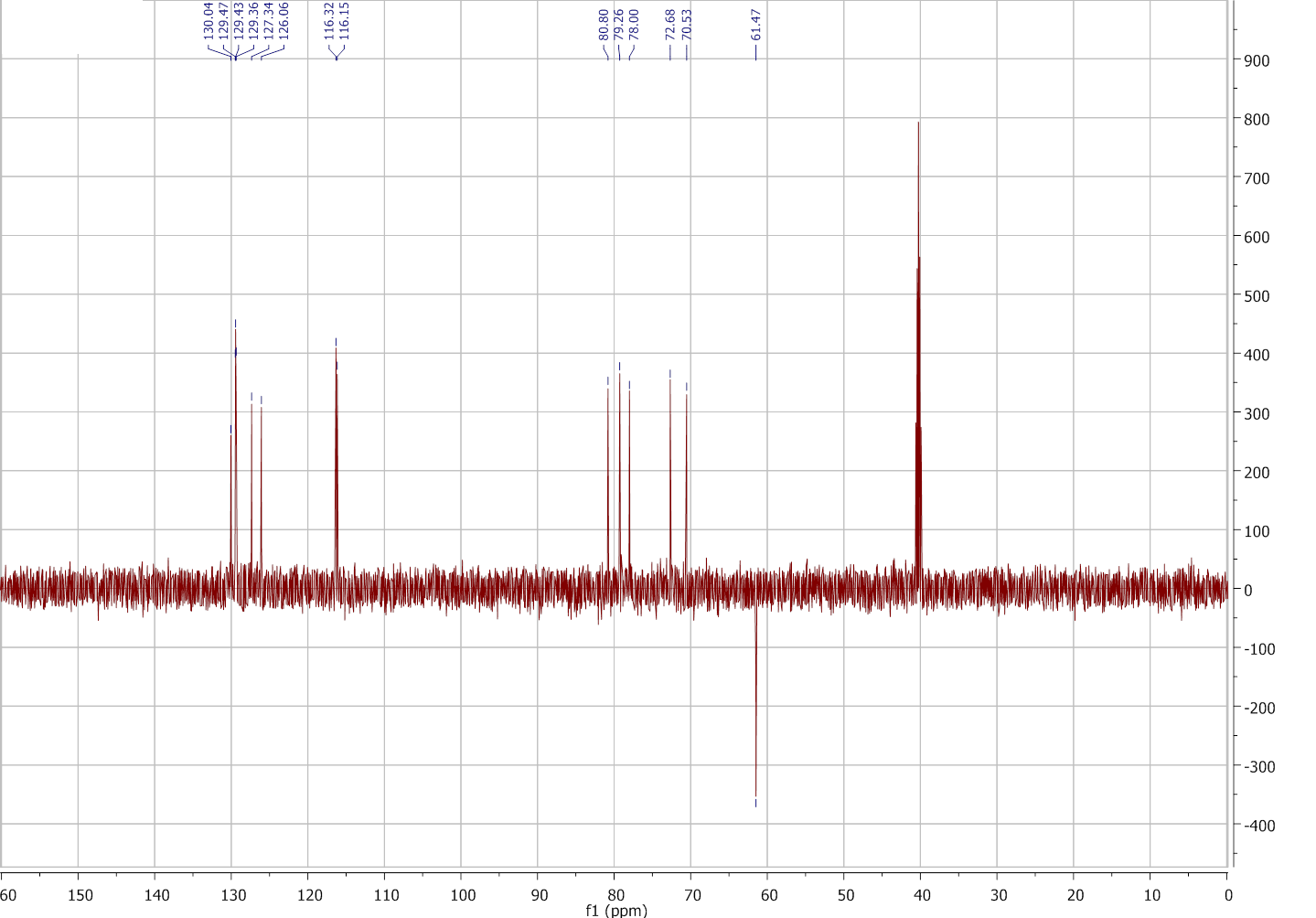
4'-fluoro-N-(3,4,5-trihydroxy-6-(hydroxymethyl)tetrahydro-2H-pyran-2-yl)biphenyl-3-carboxamide (**3**; ZHAWOC6072)

LC-MS


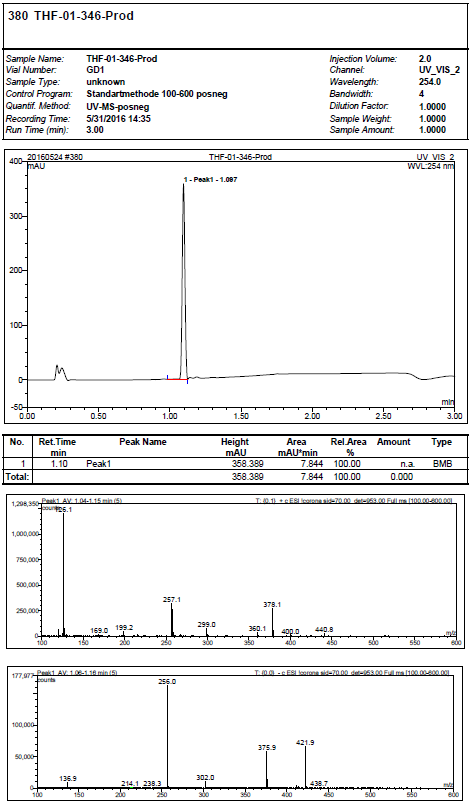


4'-fluoro-N-(3,4,5-trihydroxy-6-(hydroxymethyl)tetrahydro-2H-pyran-2-yl)biphenyl-3-carboxamide (**3**; ZHAWOC6072)

HPLC


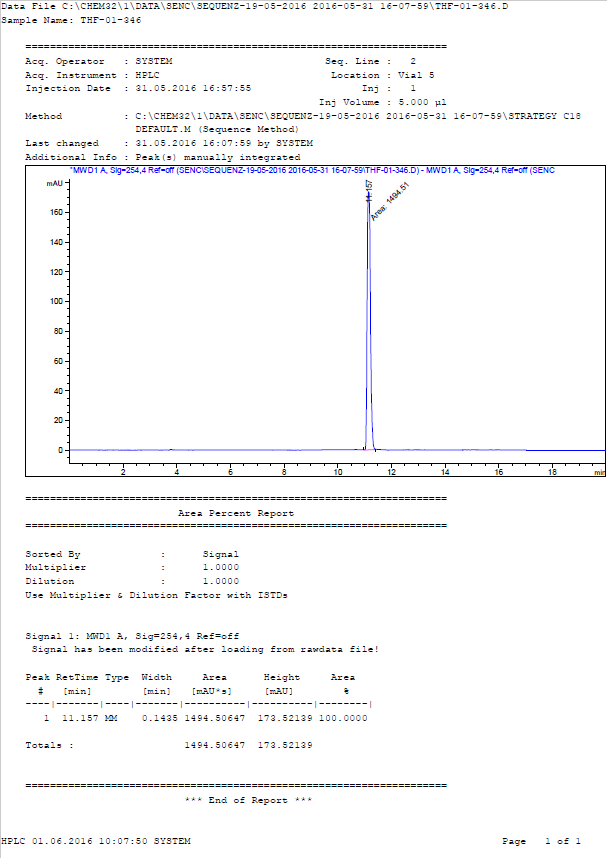


N-(3,4,5-trihydroxy-6-(hydroxymethyl)tetrahydro-2H-pyran-2-yl)biphenyl-4-carboxamide (**4**; ZHAWOC6077)

NMR


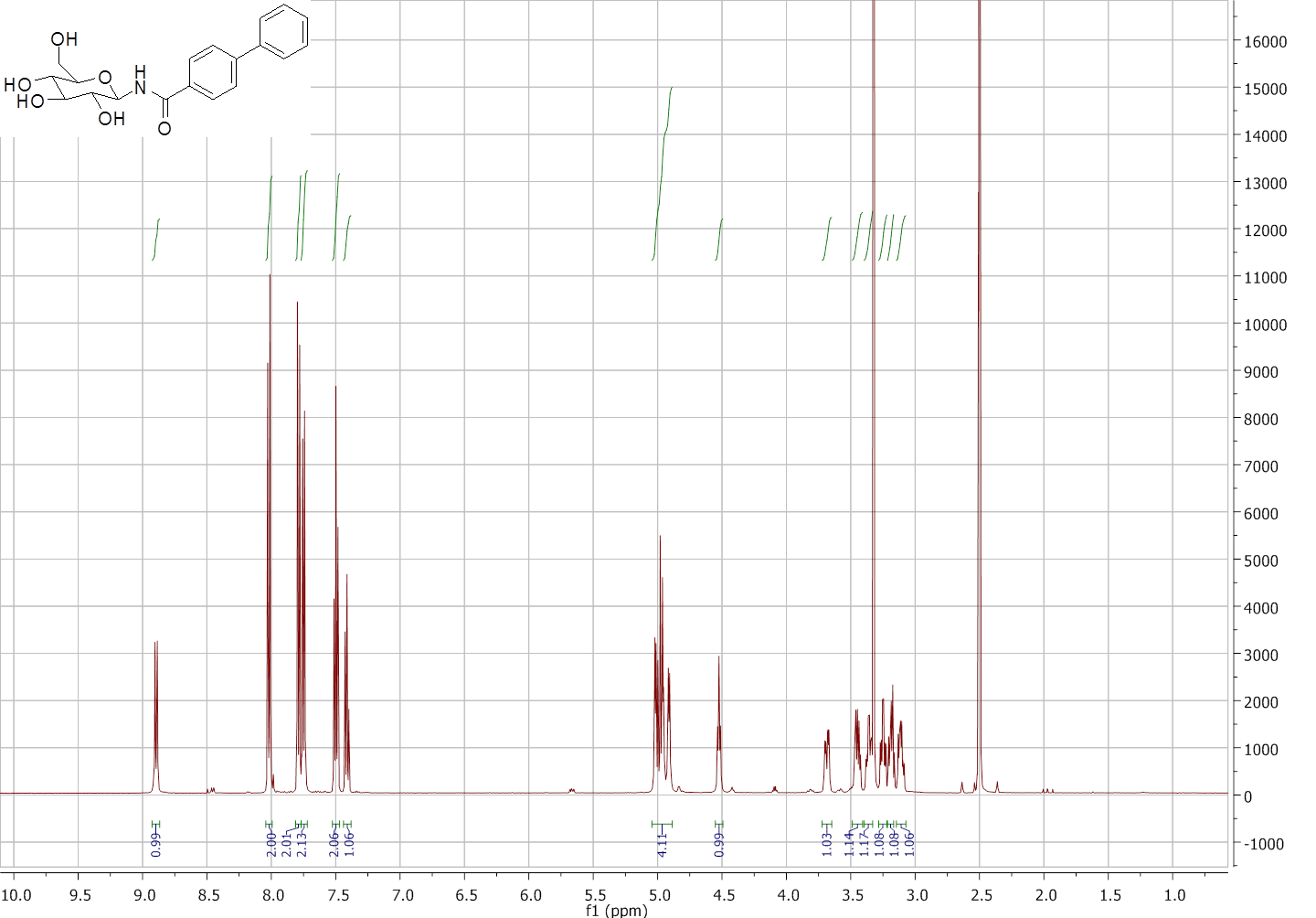


N-(3,4,5-trihydroxy-6-(hydroxymethyl)tetrahydro-2H-pyran-2-yl)biphenyl-4-carboxamide (**4**; ZHAWOC6077)


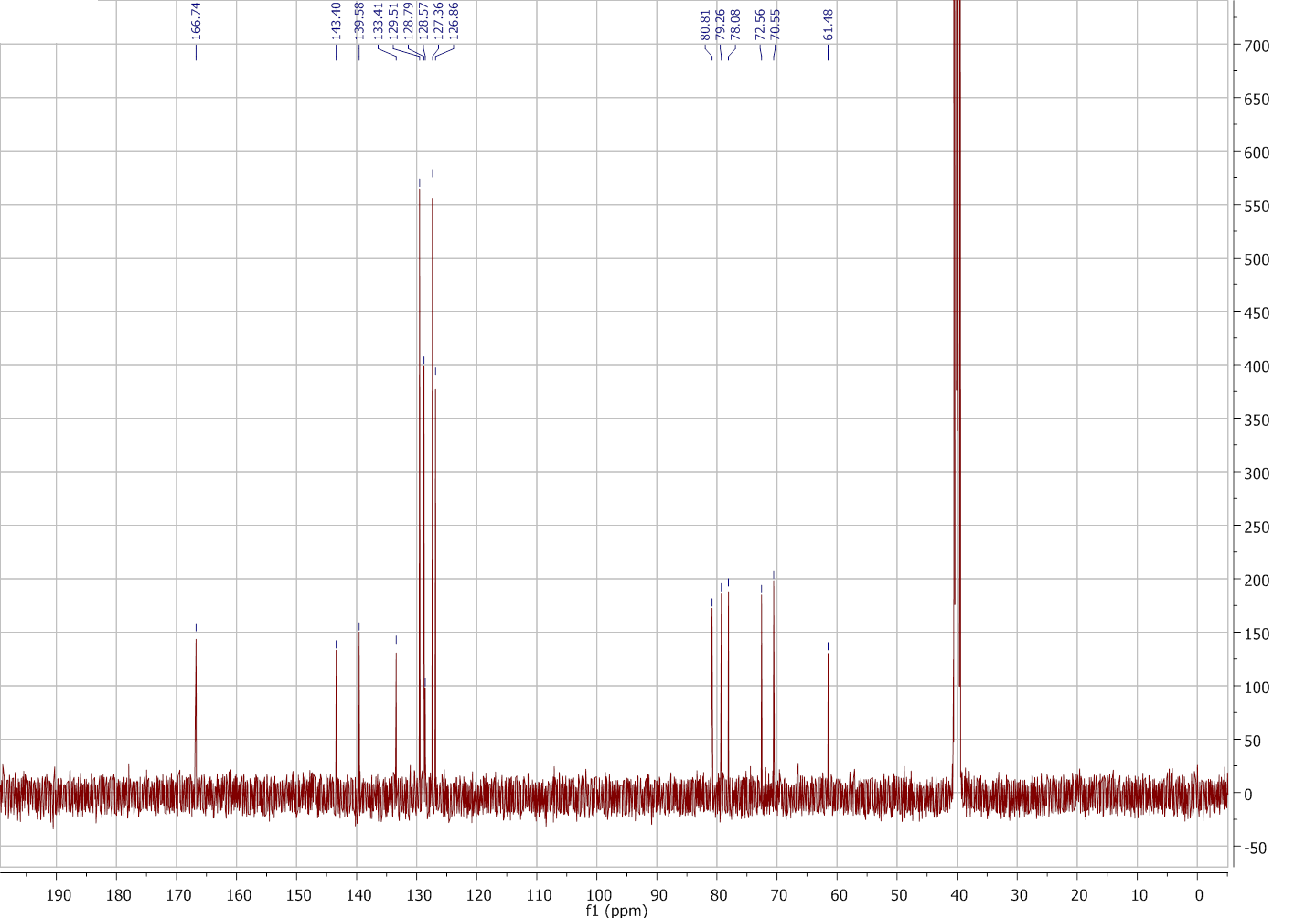


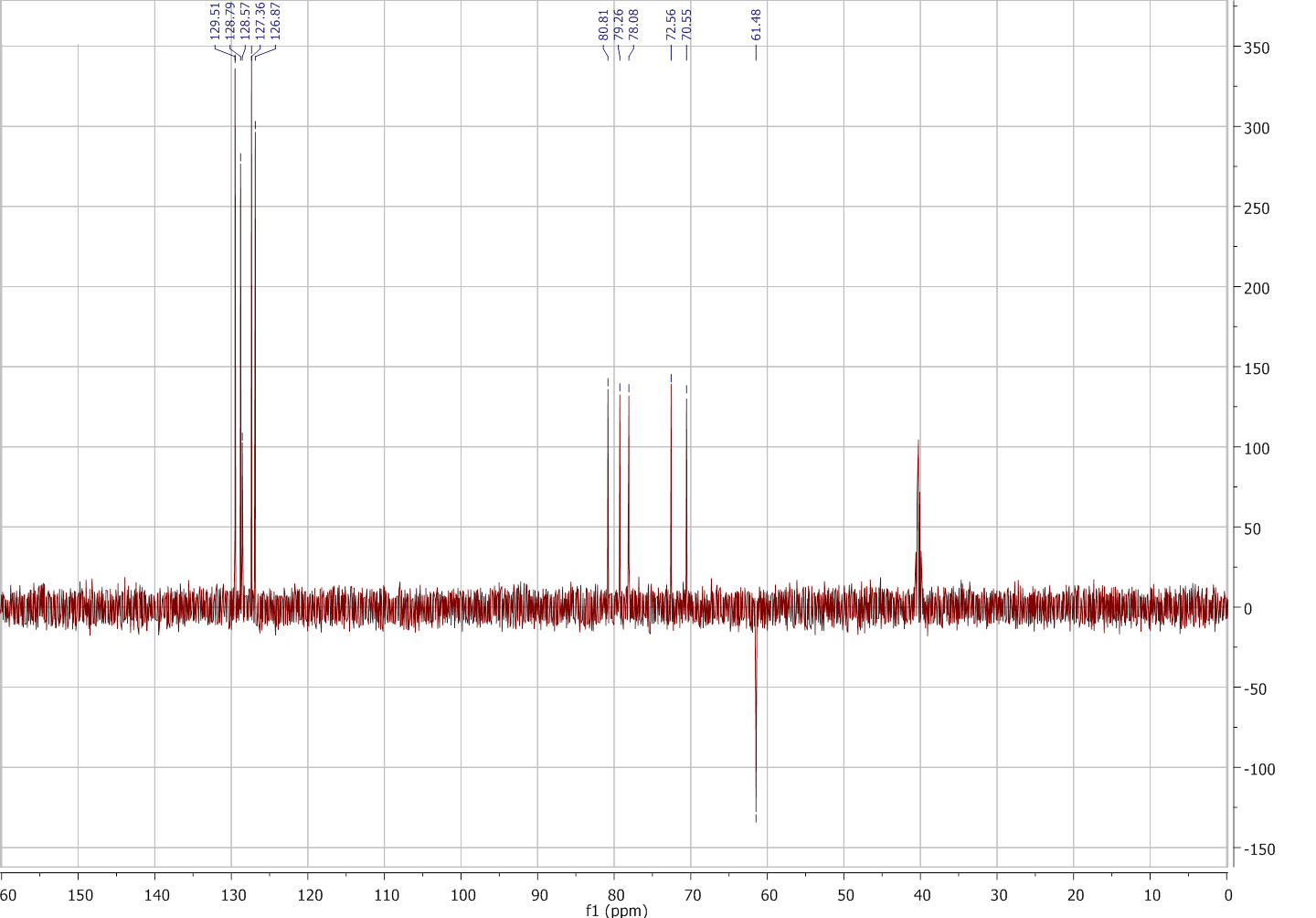


N-(3,4,5-trihydroxy-6-(hydroxymethyl)tetrahydro-2H-pyran-2-yl)biphenyl-4-carboxamide (**4**; ZHAWOC6077)

LC-MS


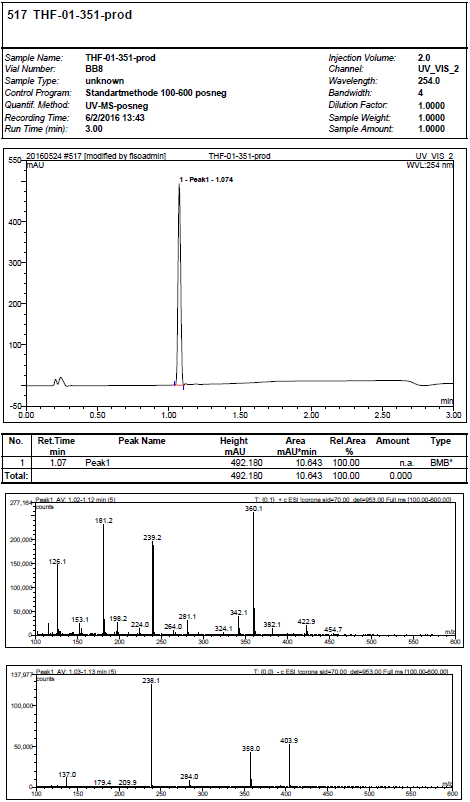


N-(3,4,5-trihydroxy-6-(hydroxymethyl)tetrahydro-2H-pyran-2-yl)biphenyl-4-carboxamide (**4**; ZHAWOC6077)

HPLC


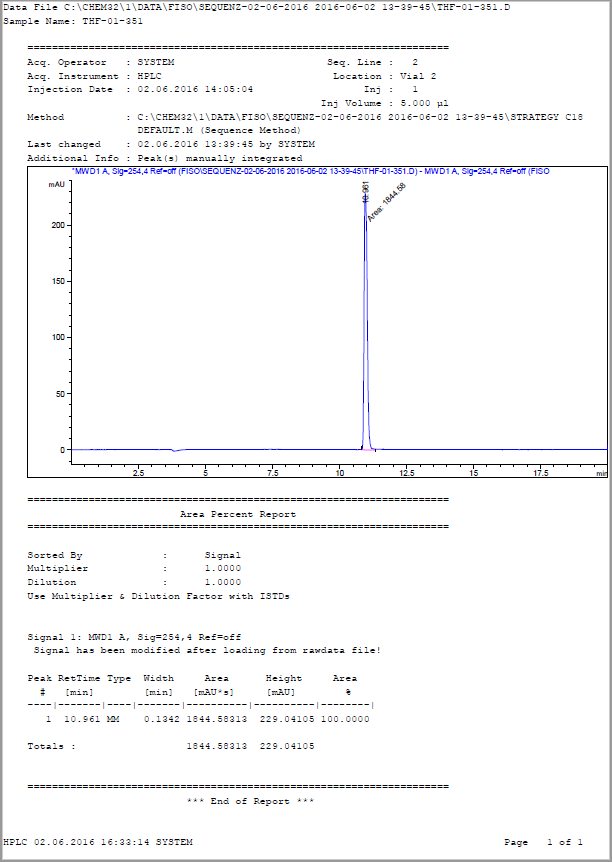


**Kinetics**

**
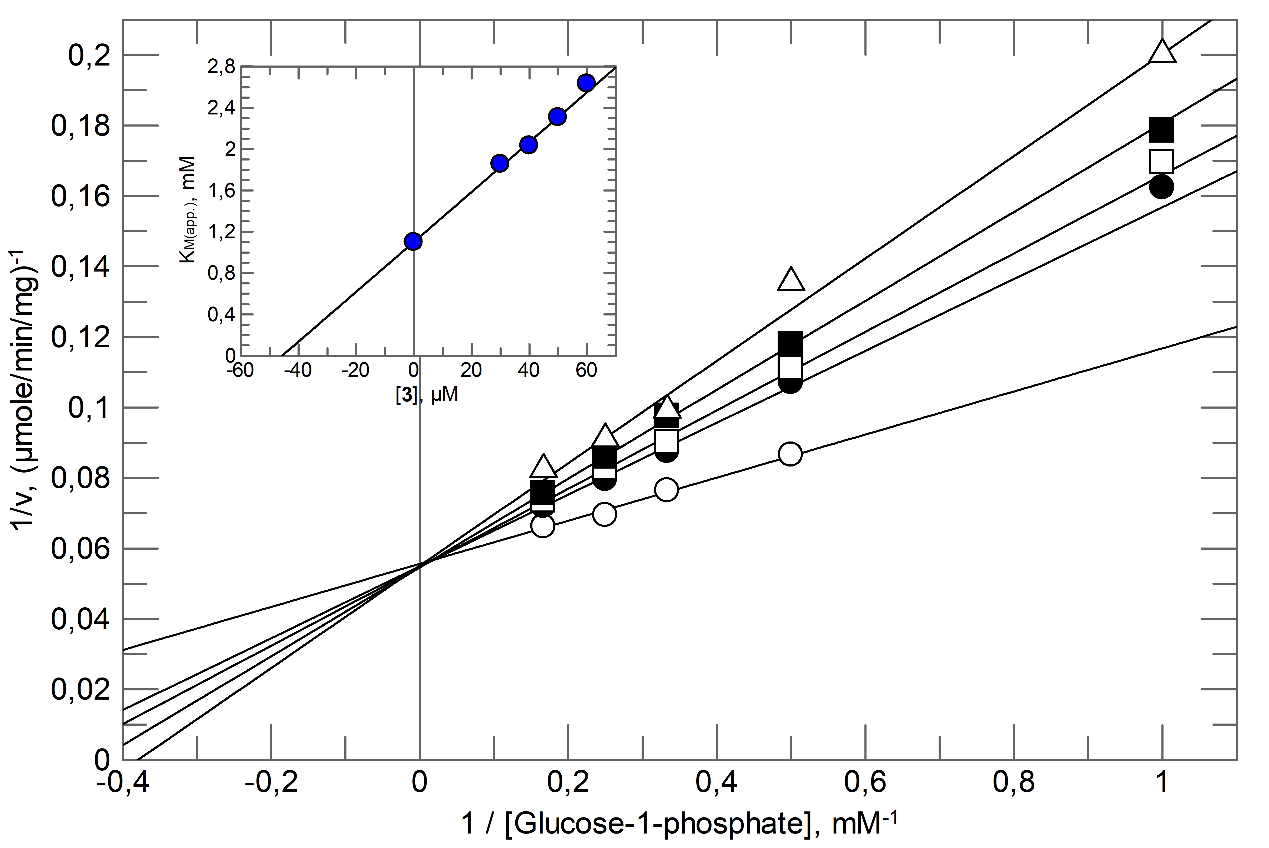
**

**
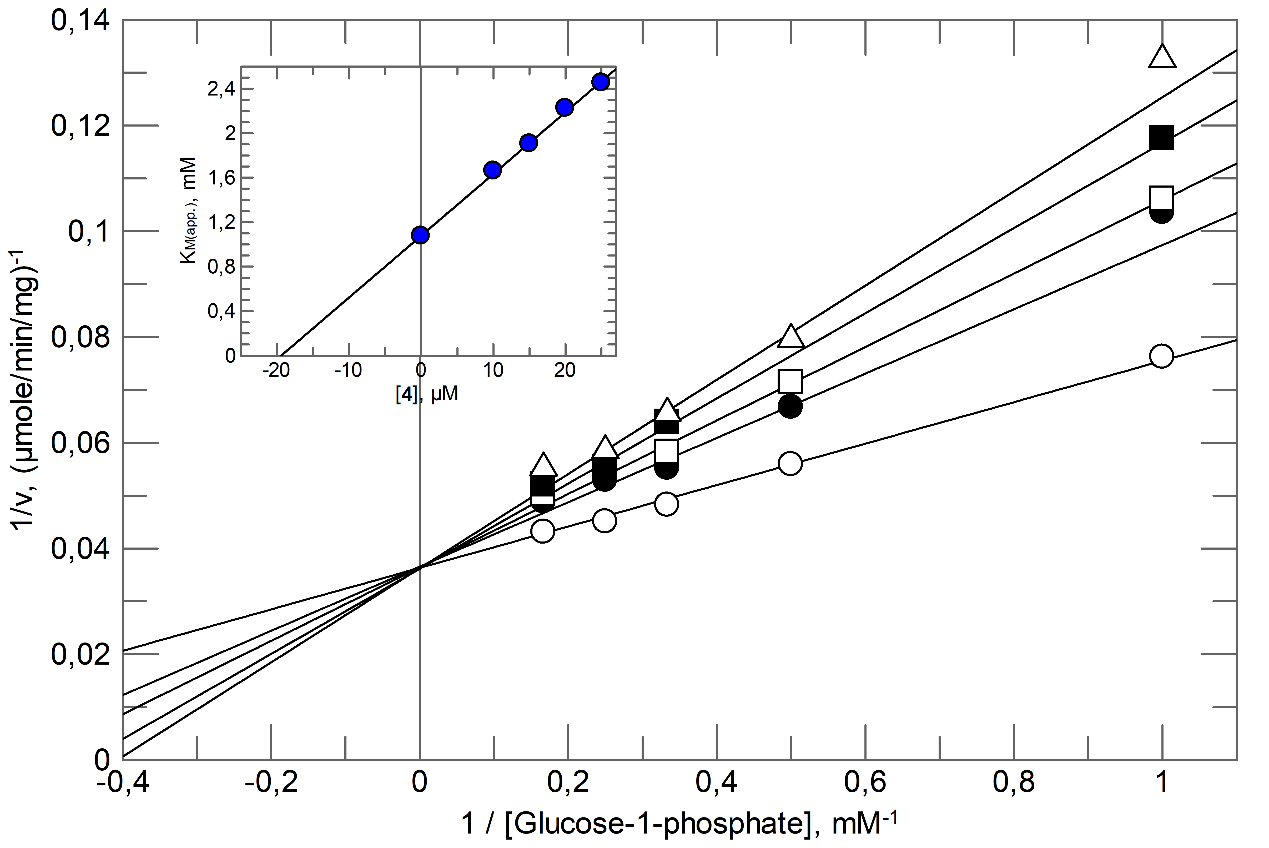
**

**Figure 1.** Competitive inhibition of hlGPa at 0.2% *w*/*v* glycogen, different Glc-1-P concentrations (2, 3, 4, 6, and 10 mM) and different inhibitor concentrations. The inset plot of the *K*_M(app.)_ versus inhibitor concentration yields the *K*_i_ values. For **3** concentrations used were 0.0 (⚪), 10 (•), 20 (☐), 30 (■), and 50 (**△**) μM, for **4** concentrations used were 0.0 (⚪), 30 (•), 40 (☐), 50 (■), and 60 (**△**) μM. In the inset plots each point (•) represents a *K*_M(app.)_ value.

**X-ray crystallography**

| **Table S1.** Summary of the diffraction data processing and refinement statistics for the rmGPb complexes. Values in parentheses are for the outermost shell. | | |
| --- | --- | --- |
| **rmGPb complex** | **3** | **4** |
| ***Data collection and processing statistics*** | | |
| Unit cell dimensions | a=b=128.3 Å, c=116.1 Å, α=β=γ=90° | a=b=128.3 Å, c=115.8 Å, α=β=γ=90° |
| Resolution (Å) | 13.71 - 2.50 | 13.82 - 2.40 |
| Outermost shell (Å) | 2.61 - 2.50 | 2.50 - 2.40 |
| Reflections measured | 120373 | 130910 |
| Unique reflections | 32589 (3644) | 36628 (3704) |
| Multiplicity | 3.7 (2.6) | 3.6 (2.6) |
| *R_symm_* | 0.110 (0.754) | 0.068 (0.451) |
| Completeness (%) | 96.1 (89.7) | 90.2 (87.0) |
| <I/σI> | 10.3 (1.6) | 12.2 (2.0) |
| *CC*^1/2^ | 0.977 (0.563) | 0.988 (0.772) |
| ***Refinement statistics*** | | |
| *R_cryst_* | 0.163 (0.262) | 0.147 (0.224) |
| *R_free_* | 0.212 (0.311) | 0.196 (0.268) |
| No of solvent molecules | 214 | 241 |
| ***r.m.s. deviation from ideality in*** | | |
| bonds (Å) | 0.010 | 0.008 |
| angles (°) | 1.4 | 1.3 |
| ***Average B factor (Å^2^)*** | | |
| Protein atoms | 19.2 | 19.5 |
| Solvent molecules | 25.2 | 26.1 |
| Inhibitor atoms | 30.0 | 22.9 |
| PDB entry | 6R0H | 6R0I |

| **Table S2**: Potential hydrogen bond interactions of inhibitors with rmGPb residues at the catalytic site in the crystal. | | | |
| --- | --- | --- | --- |
| **Inhibitor**  **atom** |  | **Distances (Å)** | |
|  | **Protein Residues (atoms)** | **3** | **4** |
| **O2**' | Asn284 (ND2) | 3.3 | 3.3 |
|  | Tyr573 (OH) | 3.0 | 3.1 |
|  | Glu672 (OE2) | 3.0 | 3.2 |
|  | Water170 (O) | 2.9 | 2.8 |
| **O3**' | Glu672 (OE2) | 2.8 | 2.8 |
|  | Ala673 (N) | 3.2 | 3.3 |
|  | Ser674 (N) | 2.9 | 3.1 |
|  | Gly675 (N) | 3.0 | 3.1 |
| **O4**' | Gly675 (N) | 2.8 | 2.8 |
|  | Water96 (O) | 2.6 | 2.7 |
| **O6**' | His377 (ND1) | 2.7 | 2.6 |
|  | Asn484 (ND2) | 2.7 | 2.7 |
| **O2** | Leu136 (N) | - | 3.0 |
|  | Water193 (O) | 2.8 | 3.2 |
| **N1** | Asn284 (ND2) | 3.2 | - |
|  | His377 (O) | 3.2 | - |
| **F1** | Asn282 (OD1) | 3.2 | - |
| **Total** |  | **16** | **14** |

| **Table S3.** van der Waals interactions of the inhibitors at the rmGPb active site | | |
| --- | --- | --- |
| Inhibitor atoms | **3** | **4** |
|  | Protein residues (atoms) | |
| C2' | His377 (O) |  |
| C3' | Glu672 (OE2) | Glu672 (OE1) |
| O3' | Glu672 (CG, CD) |  |
| C4' |  | Gly675 (N) |
| O4' | Ser674 (CB), Gly675 (CA, C) | Ser674 (CB), Gly675 (CA, C) |
| C5' | Gly135 (CA, C) | Gly135 (CA, C), Leu136 (N) |
| C6' | Gly135 (C), His377 (ND1, CE1,) His484 (ND2) | Gly135 (C), Leu136 (CA), Leu139 CD2), His377 (ND1, CE1), His484 (ND2) |
| O6' | His377 (CE1) | His377 (CE1) |
| C2 | Leu136 (CB), Asn284 (ND2) | Leu136 (CB) |
| O2 | Leu136 (CB) | Leu136 (CB) |
| C3 | Leu136 (CD1), Asn284 (CG, ND2) | Leu136 (CB, CD1) |
| C4 | His377 (CB), Thr378 (CG2) | Leu136 (CD1) |
| C5 | Asp339 (CG), Thr378 (CG2) | His341 (CE1) |
| C6 | His341 (CE1, NE2) | His341 CE1) |
| C7 | His341 (CE1) | Asn282 (O), Asp283 (CA), Asn284 (N) |
| C9 | His341 (CE1) | His341 (CE1, NE2) |
| C10 | His341 (CE1) | Ala383 (O) |
| C11 | Asn282 (ND2, O), Phe285 (O) | Phe285 (C, O), Phe286 (CA) Ala383 (O) |
| C12 | Asn282 (C, O, ND2), Arg292 (NH2) | Phe285 (C, O) Phe286 (CA) |
| C13 | Glu88 (OE2), Asn133 (ND2) | Arh292 (NH2), His341 (CE1, NE2) |
| C14 | Glu88 (OE2), His341 (CE1) | His341 (CE1, NE2) |
| **Total** | **40** | **40** |

**
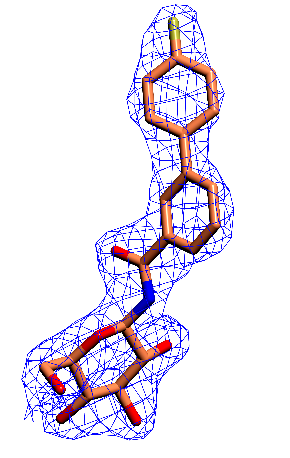

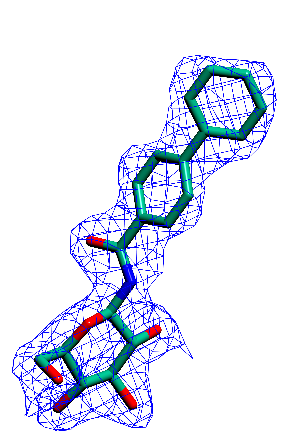
**

**Figure S2**. The REFMAC weighted 2Fo-Fc electron density maps of the bound ligands (3 and 4) at the catalytic site. Maps are contoured at 1.0 σ before the incorporation of the ligand molecules in the refinement process and the final models of the inhibitors are shown.

**Figure S3.** A movie of the superposition of the rmGPb-**3** complex (brown) onto the rmGPb-**4** complex (green) showing the different conformations of the 280s loop.
