# Supplementary figures and images for: High Consistency of Structure-Based Design and X-Ray Crystallography: Design, Synthesis, Kinetic Evaluation and Crystallographic Binding Mode Determination of Biphenyl-N-acyl-β-d-Glucopyranosylamines as Glycogen Phosphorylase Inhibitors
Source: Molecules. 2019 Apr 3;24(7):1322. doi: 10.3390/molecules24071322 (PMC6479789; doi:10.3390/molecules24071322)

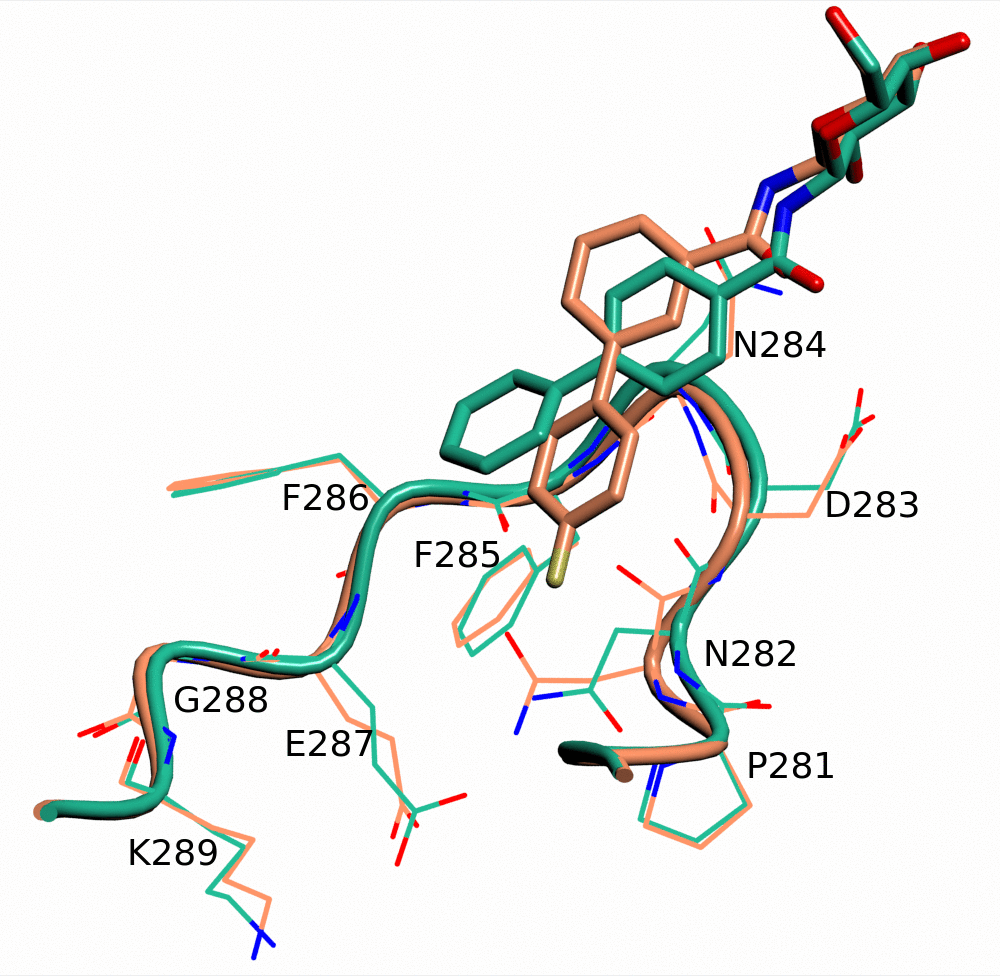

Supplement: Supplementary file 1 [file molecules-24-01322-s001.zip › sup/Movie_Figure S3_6077_6072_Loop.gif]
